# Effects of Dance Movement Therapy and Dance on Health-Related Psychological Outcomes. A Meta-Analysis Update

**DOI:** 10.3389/fpsyg.2019.01806

**Published:** 2019-08-20

**Authors:** Sabine C. Koch, Roxana F. F. Riege, Katharina Tisborn, Jacelyn Biondo, Lily Martin, Andreas Beelmann

**Affiliations:** ^1^Department of Creative Arts Therapies and Therapy Sciences, Alanus University, Alfter, Germany; ^2^School of Therapy Sciences, SRH University Heidelberg, Heidelberg, Germany; ^3^Department of Psychology, Friedrich-Schiller-University, Jena, Germany; ^4^Department of Psychology, University of Bochum, Bochum, Germany; ^5^Department of Creative Arts Therapies, Drexel University, Philadelphia, PA, United States

**Keywords:** dance movement therapy, dance interventions, meta-analysis, randomized controlled trial, clinical controlled trial, creative arts therapies, integrative medicine

## Abstract

**Background:** Dance is an embodied activity and, when applied therapeutically, can have several specific and unspecific health benefits. In this meta-analysis, we evaluated the effectiveness of dance movement therapy^1^(DMT) and dance interventions for psychological health outcomes. Research in this area grew considerably from 1.3 detected studies/year in 1996–2012 to 6.8 detected studies/year in 2012–2018.

**Method:** We synthesized 41 controlled intervention studies (*N* = 2,374; from 01/2012 to 03/2018), 21 from DMT, and 20 from dance, investigating the outcome clusters of quality of life, clinical outcomes (with sub-analyses of depression and anxiety), interpersonal skills, cognitive skills, and (psycho-)motor skills. We included recent randomized controlled trials (RCTs) in areas such as depression, anxiety, schizophrenia, autism, elderly patients, oncology, neurology, chronic heart failure, and cardiovascular disease, including follow-up data in eight studies.

**Results:** Analyses yielded a medium overall effect (*d*^2^ = 0.60), with high heterogeneity of results (*I*^2^ = 72.62%). Sorted by outcome clusters, the effects were medium to large (*d* = 0.53 to *d* = 0.85). All effects, except the one for (psycho-)motor skills, showed high inconsistency of results. Sensitivity analyses revealed that *type of intervention* (DMT or dance) was a significant moderator of results. In the *DMT cluster*, the overall medium effect was small, significant, and homogeneous/consistent (*d* = 0.30, *p* < 0.001, *I*^2^ = 3.47). In the *dance intervention cluster*, the overall medium effect was large, significant, yet heterogeneous/non-consistent (*d* = 0.81, *p* < 0.001, *I*^2^ = 77.96). Results suggest that DMT decreases depression and anxiety and increases quality of life and interpersonal and cognitive skills, whereas dance interventions increase (psycho-)motor skills. Larger effect sizes resulted from observational measures, possibly indicating bias. Follow-up data showed that on 22 weeks after the intervention, most effects remained stable or slightly increased.

**Discussion:** Consistent effects of DMT coincide with findings from former meta-analyses. Most dance intervention studies came from preventive contexts and most DMT studies came from institutional healthcare contexts with more severely impaired clinical patients, where we found smaller effects, yet with higher clinical relevance. Methodological shortcomings of many included studies and heterogeneity of outcome measures limit results. Initial findings on long-term effects are promising.

## Introduction

### Why This Meta-Analysis?

Dance movement therapy (DMT) is the psychotherapeutic use of movement, based on the assumption of the interconnection of body and mind, and the healing power of dance. The American Dance Therapy Association (ADTA) defines it as “the psychotherapeutic use of movement to promote emotional, social, cognitive, and physical integration of the individual, for the purpose of improving health and well-being” (ADTA, 2018); the European Association Dance Movement Therapy adds the “spiritual integration” to this list (EADMT, 2018). At the present state of professionalization, next to the development of knowledge on mechanisms of DMT (Koch, 2017), and of arts-based research methods (Hervey, 2000; Leavy, 2017), the implementation of evidence-based research is crucial for the survival, recognition, and thriving of the clinical field of DMT (see, e.g., Wengrower and Chaiklin, 2008; Bräuninger, 2012a,b; Dunphy et al., 2019) in the worldwide healthcare systems.

While the use of dance as a healing art is presumably as old as mankind, DMT became an established profession in Western countries from the 1940s, when the first pioneers developed professional dance therapy concepts, which spread in Eastern countries and worldwide beginning in the 1990s. Most DMT professionals work in psychiatric hospitals, rehabilitation centers, schools, and private practice. Throughout its existence, there has been much qualitative research in DMT (see, e.g., Goodill, 2005; Cruz and Berrol, 2012). However, in recent years, evidence-based quantitative research is getting stronger, in terms of numbers and quality of studies (e.g., Peters, 2012). A more general empirical evidence-base on the effectiveness of DMT will provide an orientation for patients, therapists, researchers, educators, and healthcare associations and influence professional and public recognition of DMT (Koch et al., 2014). According to standards of evidence-based medical practice, there are five levels of evidence (from the bottom to the top level): Level 4: expert opinions/textbooks, Level 3: case studies/non-experimental studies, Level 2: quasi-experimental studies, Level 1b: randomized controlled studies, and Level 1a: meta-analyses/reviews (e.g., Sackett et al., 2000). The present meta-analysis is an update of the meta-analysis published by Koch et al. (2014), tackling high-quality evidence-based research in the field. The aim was to synthesize data from controlled intervention studies published worldwide between January of 2012 and March of 2018.

Since dance is an important therapeutic ingredient in DMT, we also included studies on the effect of *dance interventions*, similar to the previous meta-analysis (Koch et al., 2014). There is a growing number of artists offering dance interventions in clinical and subclinical contexts (see Martin et al., 2018). Including dance intervention studies in addition to DMT studies had the further advantage that we obtained enough data to conduct sensitivity analysis. Dance interventions in this context are practices of various dance styles (e.g., ballroom dance, folk dance, contemporary dance), which aim to improve the quality of life or other health-related psychological outcomes of the participants. It is possible to synthesize dance intervention and DMT intervention studies, because they presumably share many *therapeutic mechanisms* (also termed *active factors*, denominating the effective ingredient of an intervention; Kazdin, 2007; Koch, 2017). Koch (2017) has distinguished five mechanism clusters through which creative arts therapies^3^ work that also apply to DMT and dance: (a) hedonism (pleasure and play, non-goal orientation), (b) aesthetic experience (experiencing beauty, body–mind unity, unity with a partner, etc.) and its authentic expression, (c) non-verbal meaning-making (communication, emotion expression and regulation, social interaction), (d) enactive transitional space (experiencing activity, agency, self-efficacy, constructive resources, test-acting, enactment, rituals, and transformation), and (e) creation (generativity, productivity; see Koch, 2017). In addition, there are mechanisms such as movement *per se* (arousal, hormonal changes, physiological changes through movement), dance *per se* (Jola and Calmeiro, 2017), as well as specific body feedback mechanisms related to distinct movement shape changes and qualities (Koch et al., 2007, 2014). Moreover, there are mechanisms of health-related changes that DMT shares with other forms of psychotherapy such as the therapeutic relationship, problem actualization, resource activation, etc. (Grawe et al., 1994; Wampold, 2015; Wampold and Irmel, 2015). Other more specific therapeutic mechanisms are connected to techniques of DMT, such as mirroring, movement analysis, non-verbal metaphors, imaginative techniques, meditative techniques, introspection, and focusing (Bräuninger, 2014). Furthermore, there are also specific group mechanisms of change. For example, if the intervention is conducted in a group setting, such mechanisms as cohesion, experience to be part of something larger, mutual trust, corrective emotional experiences, empowerment, mutual support, probing social roles, and enactive interpersonal learning are important (Schmais, 1985, 1998; Yalom, 1985; Rutan and Stone, 2001). More research is needed to deepen and sharpen the knowledge on therapeutic mechanism of DMT and dance interventions, and in turn improve outcome research in both fields.

The guiding questions and aim of this meta-analysis are to shed light on (a) the *extent* in which DMT and dance interventions initiate desired changes in health-related psychological outcomes, (b) the *moderators* or study characteristics that contribute to the variation of the effect sizes, and (c) the longevity or *duration* of these changes.

### State of Research

In this section, we will provide an overview on the *secondary trials* (meta-analyses/systematic reviews) and some general information on the *primary trials* conducted on effects of DMT and dance between January 2012 and March 2018.

#### Overview of Meta-Analyses and Reviews (Secondary Trials)

##### Four general meta-analyses up to 2014

The first general meta-analysis on DMT by Ritter and Graff Low (1996) provided a broad overview on the effects of DMT on health-related outcomes, incorporating 23 primary studies up to the year 1995. It yielded promising results about the effectiveness of DMT across various populations and diagnoses (children; adults; elderly; non-clinical, subclinical, and clinical populations; physical and psychiatric disorders), particularly improved anxiety symptoms. Furthermore, the authors reported health-improving changes in psychological conditions, movement, body-awareness, anger, and self-concept. Nevertheless, the study by Ritter and Graff Low (1996) had some limitations. The methodological quality of the incorporated studies varied considerably, and the authors did not report any analyses of heterogeneity (see critique of Cruz and Sabers, 1998).

Koch et al. (2014) replicated most of these findings. In their general meta-analysis on the effectiveness of DMT and dance interventions, they incorporated 23 primary studies published between 1995 and 2012. Results suggested that DMT and dance interventions improve anxiety levels [post-value comparison of standard mean differences (SMD; for a list of statistics and symbol abbreviation, see Appendix A), taking into account the confidence interval (CI): SMD = 0.44, CI = 0.15–0.72], depression (SMD = 0.36, CI = 0.17–0.56), well-being (SMD = 0.30, CI = 0.07–0.53), quality of life (SMD = 0.37, CI = 0.18–0.55), and body image (SMD = 0.27, CI = –0.04–0.57). They also found a positive effect for interpersonal competence (SMD = 0.45, CI = 0.07–0.83), but this effect was inconsistent across studies (*I*^2^ = 52%). The results of this study ought to be interpreted with caution due to several methodological constraints of the primary trials, small analysis clusters (four to eight studies per outcome cluster), and broad confidence intervals.

In sum, we found 11 meta-analyses and nine reviews on effects of dance and DMT, published after the last literature search in 2012 of Koch et al. (2014) up to March 2018. Only one of these publications was a more general overview on the effects of DMT on depression, anxiety, and well-being. It was a Master thesis by Peters (2012), incorporating 26 randomized or quasi-randomized trials. The results indicated that dance/DMT had small, but significant and positive effects on general well-being (*d* = 0.29, *I*^2^ = 38.65%), depression (*d* = 0.33, *I*^2^ = 42.04%), and anxiety (*d* = 0.31, *I*^2^ = 2.97), corroborating with the findings of Koch et al. (2014). The effects for general well-being and depression were moderated by *type of therapist* (with specialized dance instructors and DMT therapists yielding larger effects than non-specialized therapists, physiotherapists, exercise instructors, or researchers), but were not moderated by *age, gender, type of dance*, or *type of country*.

##### Eighteen specific (disease-related) reviews

The remaining 18 reviews and meta-analyses on effects of DMT and dance interventions focused on patient samples with specific diagnoses: seven on Parkinson's disease, four on anxiety and depression, four on physiological impairments, three on dementia/elderly, and two on oncology. The reviews on *Parkinson's disease* investigated the effects of DMT and dance on motor function and quality of life (De Dreu et al., 2012; Kiepe et al., 2012; Hackney and Bennett, 2014; Sharp and Hewitt, 2014; Loetzke et al., 2015; Shanahan et al., 2015; Aguiar et al., 2016). Significant improvements in balance (Berg Balance Scale), motor impairments (UPDRS-Scale, Timed-Up-and-Go scale), and quality of life were found in the intervention groups. Some of the effects remained significant, when comparing the results to a control group. We also found studies that yielded no improvements in quality of life and motor function (e.g., freezing of gait). Most trials reported participants' satisfaction and high adherence to the treatment. The secondary trials on *anxiety* and *depression* reported effects of DMT and dance on both outcomes including psychological distress (Kiepe et al., 2012; Peters, 2012; Boehm et al., 2014; Meekums et al., 2015). A high-quality primary trial was the study by Jeong et al. (2005) on health improvements through DMT in adolescent girls with mild depression. It showed increasing plasma serotonin concentration and decreasing concentration of dopamine in the participants of the DMT intervention group compared to participants of the control group. The effects on *medical conditions* such as heart failure and hypertension were investigated by Conceição et al. (2016), Gomes Neto et al. (2014), Kiepe et al. (2012), and Rodrigues-Krause et al. (2016). The results indicate that DMT (in this context termed “medical DMT”; Goodill, 2005) and dance might improve physiological conditions (e.g., systolic and diastolic blood pressure, V02-peak, exercise capacity) and quality of life, but not beyond the effects of other exercise interventions. One review on *dementia* and DMT in care homes showed that problematic behaviors decreased and social interaction and enjoyment in residents and care staff increased; adverse effects were also acknowledged, for example, from the care staff's perspective, there were fears of over-attachment with residents or embarrassment with dancing and concerns about staff shortages affecting the organization of regular dance sessions in the home (Guzmán-García et al., 2013). Some residents showed signs of confusion, irritability, and anxiety during the dancing (Palo-Bengtsson and Ekman, 1997; Palo-Bengtsson et al., 1998). The meta-analysis on DMT and dementia by Karkou and Meekums (2017) did not include any studies, because no trials met the inclusion criteria (i.e., randomized controlled trial led by dance movement therapist). Regarding the effects of DMT and dance on quality of life in *cancer patients*, we found mixed results. One meta-analysis on DMT in oncology reported significant standard mean differences (SMD) for the reduction of anxiety (Boehm et al., 2014; see also Archer et al., 2015). The meta-analysis on DMT in oncology reports effects on quality of life, but no evidence for effects on depression, anxiety, stress, fatigue, or body image (Bradt et al., 2011, 2015).

Because of the scarcity or low quality of evidence-based research in the field of DMT, most secondary studies (particularly the high quality Cochrane reviews) included only a few studies (between one and three studies). Besides that, most authors reported that their results did not have sufficient statistical power, because of methodological constraints of the primary trials. Nevertheless, in previous quantitative research, we can observe a pattern that DMT and dance interventions are as effective as traditional psychotherapy in various populations and conditions (Beelmann and Heinrichs, 2015). In addition, some high-quality qualitative research suggests that DMT and dance are beneficial supportive treatments to traditional care and have some specific advantages, such as the non-verbal approach, body-oriented treatment process, etc. (McNiff, 1993; Barba et al., 1995; Hervey, 2000; Levine and Land, 2016).

#### Information on Primary Trials

##### Included studies

All included trials are displayed in Table 1 in the results section and are marked with an “^*^” in the reference section. They were at least controlled intervention studies. We distinguished between primary studies in DMT and primary studies in dance interventions, and found 21 studies on the effects of DMT and 20 studies on the effects of dance on health-related outcomes (see Table 1 for populations, criteria, and results).

**Table 1:** Study characteristics chart of the included studies.

**ID**	**Title**	**References**	**Country**	***N (Pre-Test)***	**Intervention, control group activity**	**Target group (Diagnosis, age range or M, SD)**	**Treatment intensity (Entire period, frequency, session hour)**	**Randomization, dropout (Percentage total)**	**Mean ES (d_i_)**
1	Dynamic neuro-cognitive imagery improves mental imagery ability, disease severity, and motor and cognitive functions in people with Parkinson's disease	Abraham et al., 2018	USA	**20** NEG = 10 NCG = 10	DMT, Active (Learning + Exercise)	Parkinson disease, Elderly (MEG = 66.4; SDEG = 12.5)	2 weeks 5 × per week 120 min	Yes, Not Reported	0.41
2	Effectiveness of a combined dance and relaxation intervention on reducing anxiety and depression and improving quality of life among the cognitively impaired elderly	Adam et al., 2016	Malaysia	**84** NEG = 44 NCG = 40	Dance (poco-poco dance), Active (Relaxation exercise)	Cognitive deficits, Elderly (M = 70.87; SD = 8.19)	6 weeks 2 × per week 60 min	No, Not Reported	1.43
3	Backing the backbones—a feasibility study of effectiveness of dance movement psychotherapy on parenting stress in caregivers of children with autism spectrum	Aithal and Karkou, 2018	India	**11** NEG = 5 NCG = 6	DMT (Indian techniques: nritta, nrutya, natya), Non-Active	Caregivers of children with ASD, Adults (28–35 years)	2 weeks 3 × per week Minutes not reported	No, 8.33%	1.80
4	Dance therapy combined with patient education improves quality of life of persons with obesity: a pilot feasibility study for a randomized controlled trial	Allet et al., 2017	Switzerland	**54** NEG = 34 NCG = 33	DMT, Active (Educational sessions)	Obesity, Adults (M = 46.19; SD = 10.15)	16 weeks 2 × per week 60 min	Quasi, 20.37%	0.38
5	Effects of dance movement therapy on selected cardiovascular parameters and estimated maximum oxygen consumption in hypertensive patients	Aweto et al., 2012	Nigeria	**38** NEG = 23 NCG = 15	DMT, Active (Educational sessions)	Hypertension, Adults (MEG = 44.1; SDEG = 12.7)	4 weeks 2 × per week 50 min	Yes, 24%	0.50
6	Effectiveness of dance in patients with fibromyalgia: a randomized, single-blind, controlled study	Baptista et al., 2012	Brazil	**75** NEG = 38 NCG = 37	Dance (Belly dance), Non-active	Fybromyalgia, Adults (18–65 years)	16 weeks 1 × per week 60 min	Yes, 6.66%	0.48
7	The effects of folk dance training on 5–6 years children's physical and social development	Biber, 2016	Turkey	**40** NEG = 20 NCG = 20	Dance (Folk Dance), Non-active (TAU)	No Diagnosis, Children (5–6 years)	8 weeks 4 × per week 40 min	No, 0%	2.32
8	Dance movement therapy group intervention in stress treatment: a randomized controlled trial (RCT) AND The efficacy of dance movement therapy group on improvement of quality of life: a randomized controlled trial	Bräuninger, 2012a,b	Germany	**162** NEG = 97 NCG = 65	DMT, Non-active	Stressed, Adults (16–65 years)	12 weeks 1 × per week 90 min	Yes, 8.02%	0.36
9	Enhancing positive affect and divergent thinking abilities: Play some music and dance	Campion and Levita, 2014	UK	**56** NEG = 15 NCG1 = 13 NCG2 = 14 NCG3 = 14	Dance, active (Quiet, listening to music, cycling)	No diagnosis Young adults (18–23 years)	One session 5 min	Yes, 7.14%	−0.17
10	Efficacy of caregiver-mediated joint engagement intervention for young children with autism spectrum disorders	Chiang et al., 2016	Taiwan	**34** NEG = 18 NCG = 16	DMT (Creative movement play) + adapted joint engagement, Non-active (TAU)	Autism Children (2–4 years)	8 weeks 2 × per week 60 min	Quasi, Not Reported	0.61
11	Creative dance improves physical fitness and life satisfaction in older women	Cruz-Ferreira et al., 2015	Portugal	**57** NEG = 32 NCG = 25	Dance (Creative dance), Non-active	No diagnosis elderly (65–80 years)	24 weeks 3 × per Week 50 min	Yes, 0%	2.54
12	Dance therapy improves motor and cognitive functions in patients with Parkinson's disease	De Natale et al., 2017	Italy	**16** NEG = 9 NCG = 7	Dance (Tango), Non-active (TAU)	Parkinson disease Elderly (M = 67; SD = 6.9)	10 weeks 2 × per week 60 min	No, 12.5%	0.25
13	Influencing self-rated health among adolescent girls with dance intervention	Duberg et al., 2013	Sweden	**101** NEG = 48 NCG = 53	Dance (African, Jazz, Contemporary), Non-active	No diagnosis Teenager (13–18 years)	32 weeks 2 × per week 75 min	Yes, Not Reported	0.51
14	Cognitive benefits of a dance movement therapy program in adults with intellectual disabilities	Guerra-Balic et al., 2017	Spain	**28** NEG = 13 NCG = 15	DMT (Chacian approach), Non-active	Intellectual disability Adults (44–66 years)	13 weeks 2 × per week 60 min	No, Not reported	0.11
15	Effects of dance on motor functions, cognitive functions, and mental symptoms of Parkinson's disease: a quasi-randomized pilot trial	Hashimoto et al., 2015	Japan	**46** NEG = 15 NCG = 14	Dance (Modern, Jazz, Ballet, Aerobic), Active (physical exercise) + Non-active	Parkinson-disease Elderly (Mean = 67.9; SD = 7)	12 weeks 1 × per week 60 min	Quasi, 21.74%	0.71
16	“We dance and find each other”: Effects of dance/movement therapy on negative symptoms in autism spectrum disorder	Hildebrandt et al., 2016	Germany	**78** NEG = 53 NCG = 22	DMT, Non-active	ASD Teenager + Adults (14–53 years)	10 weeks 1 × per week 60 min	Yes, 57.38%	0.24
17	Effects of a short-term dance movement therapy program on symptoms and stress in patients with breast cancer undergoing radiotherapy: a randomized, controlled, single-blind trial	Ho et al., 2016a	Hong Kong	**139** NEG = 69 NCG = 70	DMT, Non-active (TAU)	Breast cancer Adults (18+years)	3 weeks 2 × per week 90 min	Yes, 10.77%	0.06
18	Effects of exercise training with traditional dancing on functional capacity and quality of life in patients with schizophrenia: a randomized controlled study	Kaltsatou et al., 2015	Greece	**31** NEG = 16 NCG = 15	Dance (Greek traditional dance), Non-active (TAU, e.g., Psycho-therapy)	Schizophrenia Adults (Mean = 59.9; SD = 14.1)	32 weeks 3 × per week 60 min	Yes, 0%	0.35
19	Six months of dance intervention enhances postural, sensorimotor, and cognitive performance in elderly without affecting cardio-respiratory functions	Kattenstroth et al., 2013	Germany	**35** NEG = 25 NCG = 10	Dance (“Agilando”-special dance for elderly), Non-active	No diagnosis Elderly (60–94 years)	24 weeks 1 × per week 60 min	Quasi, Not Reported	0.65
20	Fixing the mirrors a feasibility study of dance movement therapy on young adults with autism spectrum disorder	Koch et al., 2014	Germany	**31** NEG = 16 NCG = 15	DMT, Non-active	Autism Young adults (16–47 years)	7 weeks 1 × per week 60 min	Quasi, 0%	0.55
21	Breaking barriers: evaluating an arts-based emotion regulation training in prison	Koch et al., 2014	Germany	**47** NEG = 29 NCG = 18	DMT based anti-violence training, Non-active	No diagnosis, Imprisoned Adults (21–63)	1 week 5 × per week 60 min	Quasi, Not Reported	0.02
22	Embodied self in trauma and self-harm: effects of a single flamenco therapy session on traumatized in-patients. A pilot study	Koch et al., 2019	Germany	**33** NEG = 16 NCG = 16	DMT (Flamenco based), Non-active (TAU)	Trauma Adults (18–59 years)	1 week 1 × per week 60 min	Quasi, 2.13%	0.54
23	Fostering social cognition through an imitation and synchronization-based dance-movement intervention in adults with autism spectrum disorder: a controlled proof-of-concept study	Koehne et al., 2016	Germany	**51** NEG = 27 NCG = 24	Dance (Synchronization based movement intervention), Active (Controlled movement intervention)	High-function ASD Young adults (M = 32.75; SD = 9.1)	10 weeks 1 × per week 90 min	No, 7.27%	0.34
24	Effectiveness of dance/movement therapy on affect and psychotic symptoms in patients with schizophrenia	Lee et al., 2015	Korea	**38** NEG = 18 NCG = 20	DMT, Non-active (TAU)	Schizophrenia Adults (M = 41.5; SD = 9.1)	12 weeks 1 × per week 60 min	Quasi, Not Reported	0.28
25	Overcoming disembodiment: The effect of movement therapy on negative symptoms in schizophrenia—A multicenter randomized controlled trial	Martin et al., 2016	Germany	**68** NEG = 44 NCG = 24	DMT, Non-active (TAU)	Schizophrenia Adults (M = 39.84; SD = 10.35)	10 weeks 2 × per week 90 min	Yes, 30.88%	0.74
26	Does 12-week Latin dance training affect the self-confidence of the university students?	Meric and Ilhan, 2016	Turkey	**60** NEG = 30 NCG = 30	Dance (Latin), Not described	No diagnosis Young adults (M = 20.4; SD = 1.99)	12 weeks 1 × per week 120 min	No, Not reported	0.66
27	Dance therapy and the public school: The development of social and emotional skills of high school students in Greece	Panagiotopoulou, 2018	Greece	**23** NEG = 11 NCG = 12	DMT, Non-active	No diagnosis Teenager (16–17 years)	12 weeks 1 × per week 60 min	No, 0%	0.29
28	Argentine tango dance compared to mindfulness meditation and a waiting-list control: A randomized trial for treating depression	Pinniger et al., 2012	Australia	**66** NEG = 21 NCG1 = 29 NCG2 = 16	Dance (Argentinean Tango), Active (physical exercise) + Non-active	Stress Adults (18–80 years)	24 weeks 1 × per week 90 min	Yes, 31.96%	0.39
29	Tango dance can reduce distress and insomnia in people with self-referred affective symptoms	Pinniger et al., 2013	Australia	**64** NEG = 24 NCG1 = 25 NCG2 = 11 NCG3 = 12	Dance Argentinean Tango Active (Meditation, Exercise), Non-active	Stress Adults (18–68)	8 weeks 1 × per week 90 min	Yes, 34.02%	0.67
30	Changes in well-being of schizophrenic patients after movement therapy: results of a multicenter RCT-study	Pohlmann et al., 2017	Germany	**36** NEG = 24 NCG = 12	DMT, Non-active	Schizophrenia Adults (18–83)	20 weeks 1 × per week 90 min	Yes, 47.06%	0.50
31	Effectiveness of group body psychotherapy for negative symptoms of schizophrenia: multicentre randomized controlled trial	Priebe et al., 2016	UK	**275** NEG = 140 NCG = 135	DMT (Manualised body psychotherapy), Active (Pilates)	Schizophrenia Adults (M = 42.2; SD = 10.7)	10 weeks 2 × per week 90 min	Yes, 4.54%	0.19
32	A dance movement therapy group for depressed adult patients in a psychiatric outpatient clinic: Effects of the treatment	Pylvänäinen et al., 2015	Finland	**33** NEG = 21 NCG = 12	DMT (Chacian approach), Non-active (TAU)	Depression Adults (20–59 years)	12 weeks 1 × per week 90 min	No, 22%	0.74
33	Tango for treatment of motor and non-motor manifestations in Parkinson's disease: A randomized control study	Rios Romenets et al., 2015	Canada	**33** NEG = 18 NCG = 15	Dance (Argentinean Tango), Active (Self-directed exercise)	Parkinson-disease Elderly MEG = 63.2; SDEG = 9.9)	12 weeks 2 × per week 60 min	Yes, 27.27%	0.20
34	An exploratory randomized controlled trial of body psychotherapy for patients with chronic depression	Röhricht et al., 2013	UK	**21** NEG = 16 NCG = 15	DMT, Non-Active (TAU)	Depression Adults (18–65 years)	10 weeks 2 × per week 90 min	Yes, 24.24%	1.16
35	Effect of dance on cancer-related fatigue and quality of life	Serrano-Guzmán et al., 2016	Spain	**67** NEG = 35 NCG = 32	DMT, Non-Active (TAU)	Hypertension Adults + Elderly (62–76)	8 weeks 3 × per week 50 min	Yes, 0%	0.55
36	Effect of dance on cancer-related fatigue and quality of life	Sturm et al., 2014	Germany	**40** NEG = 20 NCG = 20	Dance (Group choreography), Non-active	Cancer Adults (26–74)	5 weeks 2 × per week 60 min	Quasi, 10%	1.03
37	Dance improves functionality and psychosocial adjustment in cerebral palsy	Teixeira-Machado et al., 2017	Brazil	**26** NEG = 13 NCG = 13	Dance, active (kinesiotherapy)	Cerebral Palsy Teenager + Young adults (15–29 years)	12 weeks 2 × per week 60 min	Yes, 0%	2.79
38	The effect of dance on depressive symptoms in nursing home residents	Vankova et al., 2014	Czech Republic	162 NEG = 79 NCG = 83	Dance (Exercise dance for seniors), Non-active (TAU)	Various diagnosis Elderly in nursing homes (60+ years)	12 weeks 1 × per week 60 min	Yes, Not reported	0.32
39	A pilot study to evaluate multi-dimensional effects of dance for people with Parkinson's disease	Ventura et al., 2016	USA	**15** NEG = 8 NCG = 7	Dance (Ballet, Musical-Jazz) + DMT Elements, active (Parkinson support group)	Parkinson Disease Elderly (MEG = 71.8; SDEG = 3.6)	10 weeks 1 × per week 75 min	No, 0%	0.61
40	A comparison of Irish set dancing and exercises for people with Parkinson's disease: a phase II feasibility study	Volpe et al., 2013	Italy	**24** NEG = 12 NCG = 12	Dance (Irish set dancing), Active (Standard physiotherapy exercises)	Parkinson- disease Elderly (56–73 years)	24 weeks 1 × per week 90 min	Yes, 0%	0.99
41	Active factors in dance/movement therapy: Specifying health effects of non-goal-orientation in movement	Wiedenhofer and Koch, 2017	Germany	**56** NEG = 28 NCG = 28	DMT (Improvisational non-goal-oriented movements)	No diagnosis Adults (19–49 years)	One session 50 min	Quasi, 1.79%	0.25

*NEG, number of subjects in the intervention group; NCG, number of subjects in the control group; M, mean; MEG, mean in intervention group; SDEG, standard deviation in intervention group; TAU, treatment as usual; ES, effect size. Bold values indicate the total sample of each study*.

##### Excluded studies

We located a number of *high-quality primary studies* that we were not able to include due to the defined criteria. Fourteen studies had been excluded due to missing data or other reasons outlined in the Methods section, some of which may be possibly recovered for future analyses. They investigated learning disabilities (Alotaibi et al., 2017), children with attention deficit/hyperactivity disorder (Alrazain et al., 2018), fornix integrity (Burzynska et al., 2017), depression (Cross et al., 2012), falls (Duim et al., 2015), oncological patients (Ho et al., 2016b), schizophrenia (Koch et al., 2017; Savill et al., 2017), Parkinson's disease (Lewis et al., 2014), autism (Mateos-Moreno and Atencia-Doña, 2013), trauma in unaccompanied minors (Meyer DeMott et al., 2017), development of kindergarteners (Stück and Villegas, 2017), personal development and increase of emotional intelligence in students (Vancea, 2013), and traumatized children (Van Westrhenen et al., 2019).

Mainly through the reviews, we also found a number of formerly undetected studies from 2011 and earlier, not yet included into the general meta-analyses. We consider it important to enumerate them here for potential future analyses: Belardinelli et al. (2008), Burgess et al. (2006), Chouhan and Kumar (2011), Connolly et al. (2011), Coubard et al. (2011), Hall (2011), Hwang et al. (2010), Kaltsatou et al. (2011, 2015), Quiroga Murcia et al. (2009), and Xiong and Li (2009).

In general, our literature search revealed that there were at least as many studies on *physiological changes* after DMT and dance interventions as there were for *psychological changes* in health outcomes in the time frame of January 2012 to March 2018. We found around 50 primary studies with mere physical/physiological outcomes, which we excluded. However, since embodiment approaches such as DMT assume the body–mind unity, we would encourage researchers to include studies focusing on physical changes into future meta-analyses in dance and DMT.

### Incorporated Outcomes

Synthesizing the outcome foci of the primary studies, this meta-analysis differentiates six outcome clusters: (a) quality of life, (b) clinical outcomes (e.g., anxiety, depression), (c) interpersonal skills, (d) cognitive skills, (e) (psycho-)motor skills, and (f) residuals (psychotic symptoms and physiological change). We based the allocation of dependent variables to outcome clusters on the meta-analysis of Koch et al. (2014) and detailed investigation of the primary trials. Our aim was to synthesize outcome clusters that were as comparable as possible (e.g., by similarity of measurement instruments).

#### Quality of Life

Quality of life is a broad construct, which contains subscales about subjective well-being (e.g., satisfaction with life) and conditions of daily living (e.g., general health, functional capacity and social integration). We decided to further include dependent variables about sleep quality, pain (invert coding), self-esteem, and control beliefs in this cluster. Most measures in this outcome cluster were based on self-report questionnaires (e.g., rating satisfaction with certain life conditions on a Likert scale), with only one study containing observations and ratings from an external person, who in this case was a clinician (Teixeira-Machado et al., 2017). We assume that DMT and dance interventions might influence quality of life on various dimensions, for example, movement might improve vitality and fitness, dance might foster joyful experiences (Koch et al., 2007), and interpersonal experiences might have a positive influence on social integration (Sandel et al., 1993).

#### Clinical Outcomes

Clinical outcomes summarize dependent variables directly related to conditions of mental health, particularly affective disorders (e.g., depression, anxiety, stress, anger). Studies in this cluster were conducted with a clinical (e.g., persons with a diagnosis of depression) or subclinical population (e.g., persons at risk for depression). Usually, the dependent variables were assessed with self-report questionnaires (e.g., HADS, BDI, BSI, STAXI). Two studies contained an interview and one study used external observation and rating. We decided to conduct a sub-analysis of anxiety and depression, because the treatment of these conditions is of broad public interest and has been discussed in the previous literature (Peters, 2012; Koch et al., 2014). We assumed that DMT and dance interventions improve psychological functions of emotion regulation, which may be mediated, for example, by authentic expression, experienced agency, body–mind integration, and physiological changes.

#### Interpersonal Skills

The term “interpersonal skills” relates to competences persons apply in social interaction (e.g., empathy, synchronization, communication, prosocial behavior, self-other awareness, maintaining a relationship). Most studies in this cluster were conducted with children, some of whom had been diagnosed with developmental disorders (ASD, ADHD). Therefore, researchers used external observations (e.g., by a parent, teacher, or clinician) more frequently. There were also two adult populations (ASD, schizophrenia). Interpersonal experiences in DMT and dance might particularly improve skills allocated to this cluster, for example, the therapeutic relationship, group cohesion, and (non-verbal) communication. In recent years, researchers have started to investigate whether the “mirroring” technique, proposed by dance movement therapist Marian Chace (Sandel et al., 1993), fosters empathy and enhances activity of mirror neurons in the brain (McGarry and Russo, 2011). A link of mirroring in movement and attachment has recently been established (see Feniger-Schaal et al., 2018, this issue).

#### Cognitive Skills

Cognitive skills relate to the set of mental abilities and processes that we need to carry out any task from the simplest to the most complex (e.g., skills of language, memory, and conceptualizing). Because a decrease of these mental abilities is a typical issue in elderly persons, most primary studies focus on this population. Cognitive skills were assessed using psychological tests (e.g., memory tasks, word tasks, calculation tasks, attention tasks) and tests referring to body image or body imagery, which denominates the ability to perceive and visualize bodily charges and changes. Since the operationalizations of the concept in the included studies measure a mainly representational skill, we categorize it under cognitive skills, even though it includes sensorimotor and emotional aspects.

#### (Psycho-)Motor Skills

The assessment of (psycho-)motor skills was conducted in (mostly elderly) patients that were diagnosed with Parkinson's disease. It contained tests on walking, turning, balance, and freezing of gait, and self-report measures on daily functioning. Only dance interventions, no DMT, were found in this outcome cluster. Dance improves motor function by training muscular activity, balance, and flexibility. Furthermore, there is a training of cognitive skills associated with movement, for example, in executing imagined movements, following music, and observing bodily changes (Hashimoto et al., 2015). Because we focused on psychological changes in this meta-analysis, studies with outcomes on mere physical skills (e.g., exercise capacity, arm range) were excluded. However, in our literature search, we found at least as many studies on physiological changes after DMT and dance interventions as on psychological changes. It is important to note that *changes in Parkinson's disease severity were categorized as psychomotor skills*, because Parkinson's disease is an extrapyramidal and neurodegenerative disorder (ICD 10), which entails physical and psychological components. The included studies with a focus on Parkinson's disease measured mixed psycho-physiological variables (as outlined above).

#### Residuals

Our residual category contained two types of outcomes too small to analyze in separate clusters: positive symptoms in schizophrenia and physiological changes (e.g., blood pressure). In schizophrenia, we distinguish positive symptoms (an overabundance of perceptions compared to average, e.g., hallucinations) from negative symptoms (a void or lack of perception and expression compared to average, e.g., apathy, mood, and blunted affect). While positive symptoms can be successfully addressed by anti-psychotic medication, DMT seems to be particularly useful for addressing negative symptoms (Röhricht and Priebe, 2006; Lee et al., 2015; Martin et al., 2016; Pohlmann et al., 2017). We allocated the negative symptoms to the clinical outcomes cluster. Pohlmann et al. (2017) postulated a concept of “disembodiment” in schizophrenia stressing that schizophrenia is a self-disorder and is characterized by disturbances of ipseity (selfhood). They state that mechanisms of body–mind integration improve self-awareness. Regarding physiological changes, the effects of dance and DMT are also detectable on a neural and hormonal level (e.g., Quiroga Murcia et al., 2009; Stück and Villegas, 2017; Abraham et al., 2018). Studies assessed positive symptoms in schizophrenia with self-report questionnaires or clinical interviews/observations, whereas physiological data were collected using medical examination procedures (e.g., sphygmomanometer).

## Methods

### Study Selection

The following inclusion criteria were used to filter studies for meta-analysis:

- Experimental intervention study (independent variable: dance or DMT intervention, dependent variable: health-related psychological outcomes)
- Control group design
- Availability of necessary statistics to calculate effect sizes (pre- and post-intervention assessment in intervention and control group, mean, SD, *N, t*, or *F* values)
- Language of publication: English or German
- Period of publication: 01/2012 to 04/2018

Because we wanted to get a broad picture, we also included studies with interventions named “body psychotherapy” or “movement integration.” We decided to classify an intervention as “DMT intervention” if a dance movement therapist conducted the session. When the qualification of therapist was missing (or unclear), we closely analyzed the descriptions of the intervention. If the intervention description suggested that predominantly typical tools of DMT were used (therapeutic use of dance/movement involving mirroring, conscious social interaction in movement, introspection and reflections on movement and body sensations; see also definition of DMT and description of DMT methods in Koch, 2019), we categorized the intervention as DMT. Trials that included dance elements but predominantly used methods of other creative arts therapies were excluded (Mateos-Moreno and Atencia-Doña, 2013; Jakobsen et al., 2017; Van Westrhenen et al., 2019). In contrast, we allocated methods to the dance intervention group, if they were dance training sessions conducted by dancers or exercise instructors from various backgrounds (physiotherapists, nurses, fitness instructors). We decided to include group as well as individual therapy sessions.

### Literature Search

For this meta-analysis, we used multiple search strategies. First, we systematically searched electronic databases, namely, Psyndex, PsycINFO, ERIC, CENTRAL, and Google Scholar. We used different terms for dance movement therapy (DMT) and dance-related interventions as single keywords and combined search terms, putting together the single keywords and terms related to the study design (see Box 1).

Box 1 **Search terms.**

**Table:** 

**Single search terms**	**Combined search terms**
• dance movement psychotherapy •dance movement therapy • dance therapy • therapeutic movement • dance-effectiveness • dance • dance-therapy • expressive movement • expressive dance	• dance movement psychotherapy + controlled trial • dance movement therapy + controlled trial • dance therapy + controlled trial • dance movement psychotherapy + random • dance movement therapy + random • dance therapy + random • dance movement psychotherapy + controlled trial • dance movement therapy + controlled trial • dance therapy + controlled trial • dance movement psychotherapy + random • dance movement therapy + random • dance therapy + random • dance movement psychotherapy + controlled trial • dance movement therapy + controlled trial • dance therapy + controlled trial • dance movement psychotherapy + random • dance movement therapy + random • dance therapy + random • dance movement psychotherapy + controlled trial • dance movement therapy + controlled trial • dance therapy + controlled trial • dance movement psychotherapy + random • dance movement therapy + random dance therapy + random

**Figure 1:**
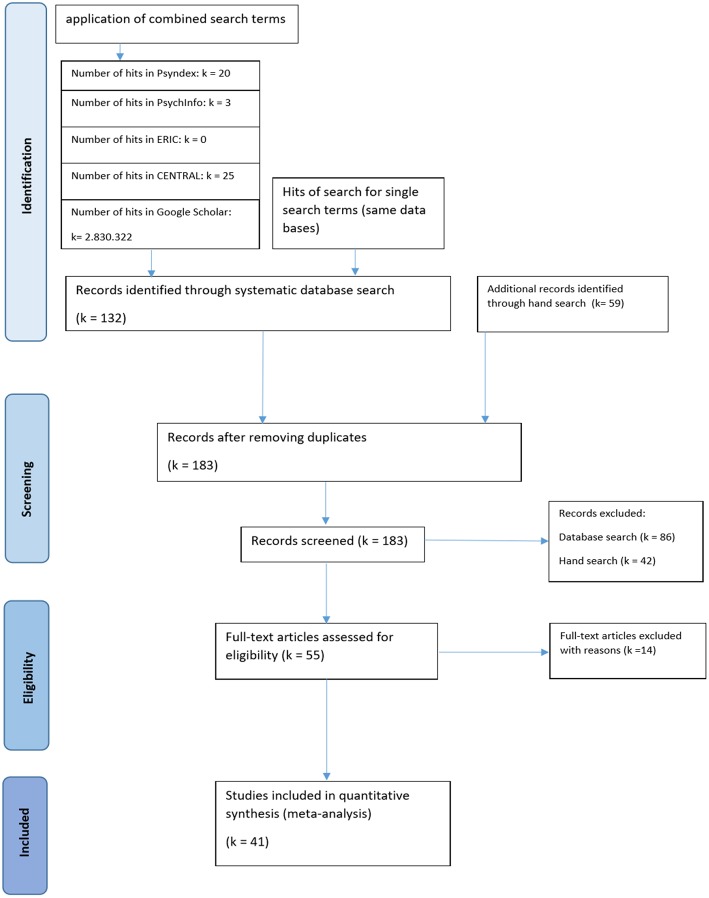
Flowchart of selection procedure. Included studies are displayed in the study characteristics overview (Table 1).

The number of hits is reported in the flowchart below (Figure 1). Additionally, we conducted a hand search examining professional journals without widespread indexing, sending requests for unpublished and in process EBM studies to national and international professional listservs, and directly wrote to researchers with a history in EBM research on dance therapy, asking for references we may have potentially missed; we also included references that were mentioned in some of the secondary studies and that slipped by our systematic literature search (we received detailed responses from Dr. Vicky Karkou, Dr. Bonnie Meekums, Dr. Iris Bräuninger, Susanne Bender, Indra Majore-Dusele, and others).

The systematic database search yielded 132 records and was supplemented with 59 studies identified through hand search. Because each included research report contained only one study, we did not have to differentiate between the analysis of research reports and studies. After removing duplicates (i.e., the same studies found in different data sources), a total of 183 studies was screened. Fifty-four of these studies (30%) met the inclusion criteria, and their full-article texts were then assessed for eligibility.

We had to exclude 14 studies for the following reasons (more information in Appendix):

- Seven studies due to insufficient data (e.g., three had no data available; for four, it was not possible to calculate reliable effect sizes from the statistics provided)
- Four studies because closer examination of the intervention methods revealed that they did not match the criteria of either DMT or dance intervention (e.g., combined creative arts therapies, observations of dance pieces)
- One study contained only qualitative data
- One study contained no comparable control group (participants of the control group were caregivers of participants with Parkinson's diagnosis in the experimental group)
- For one study, data were reported and included from another publication

Finally, we were able to include a total of 41 studies into the analysis (see Figure 1).

### Coding Procedure

We coded the study characteristics (see Table 1) using the following variables:

- *Identification:* author, title, year
- *Publication:* country, publication status
- *Sample:* sample size, age range, percentage of female participants, clinical vs. non-clinical sample, diagnosis
- *Intervention:* DMT or dance, qualification of implementing person, quality of intervention description (major intervention methods of DMT see Koch, 2019)
- *Control Group:* number of control groups, type of control group activity
- *Time:* one session vs. process, length of intervention period, frequency of intervention, length of session, period follow-up
- *Methods:* type of measurement, type of analysis, randomization, dropout

### Data Synthesis

**Figure 2:**
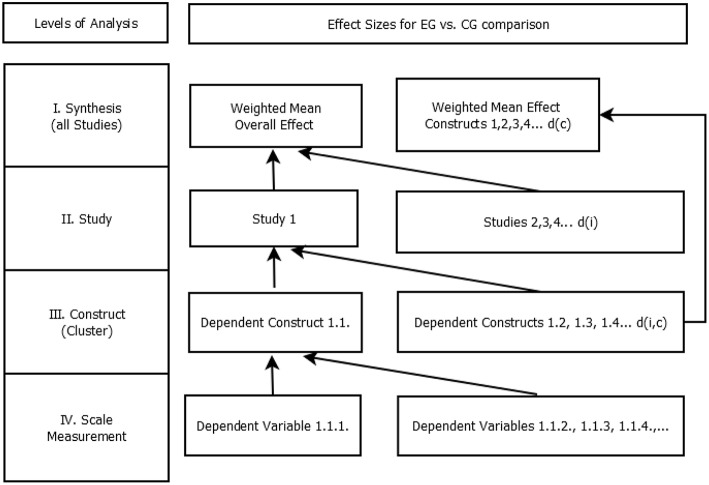
Hierarchy of effect sizes. EG, experimental group; CG, control group; d(c), weighted mean effect size per cluster; d(i), weighted mean effect size per study; d(i,c), weighted mean effect size per cluster within one study.

The analysis was done in SPSS (IBM, Version 25), employing meta-analysis macros by Wilson (2005). To synthesize data, we decided to use calculations of effect sizes. There were four levels of analysis (see Figure 2).

Most studies were using multiple scales or tests to measure the changes from pre- to post-intervention in one dependent construct (e.g., mental health, general health, and vitality as facets of quality of life). Furthermore, usually more than one dependent construct was observed in one study (e.g., quality of life, affect, and interpersonal skills). Thus, we had to synthesize data on several levels (Figure 2). From Level IV to Level III and from Level III to Level II effect sizes within studies were synthesized. From Level II to Level I effect sizes across studies were synthesized. We started our analysis at the bottom level (Level IV), calculating effect sizes for each dependent variable (e.g., scales, tests). An adjusted formula of Cohen's *d* was used (Cohen, 1988):

$$\begin{array}{l} \;d=\frac{\left(M_{IGpost}-M_{CGpost}\right)-\left(M_{IGpre}-M_{CGpre}\right)}{SDpooledpre}\\ SD_{pooled}=\sqrt{\frac{\left(N_{IGpre}-1\right)SD_{IGpre}^{2}+\left(N_{CGpre}-1\right)SD_{CGpre}^{2}}{N_{IGpre}+N_{CGpre}-2}} \end{array}$$

This formula incorporates differences between pre- and post-intervention values in the intervention group controlling for changes that occurred in the control group (for abbreviations, see list of symbols in Appendix). The effect is standardized using the pooled standard deviation, which is formed by the pooled variance of intervention and control group at the pre-intervention level. The natural variance of characteristic values in one sample is assumed to be confounded by the intervention; this is why the pooled variances at pre-time are more precise estimates of variation (Lipsey and Wilson, 2001).

Some studies reported median instead of mean and standard error of the mean or interquartile range instead of standard deviation. In these cases, we used the median as a mean and recalculated dispersion measures to approximate the effect sizes (Higgins and Green, 2008). In addition, we adjusted the polarization of the scales multiplying with −1, where necessary. Where the polarization of scales or tests remained unreported in the original study, we conducted a literature search to ascertain the direction of the effect. All effects were polarized to ensure that a positive effect size means that the health or resources of the participant improved (e.g., more interpersonal skills, less depression). In total, we calculated 306 effect sizes ranging from *d* = −0.82 to a maximum of 9.61. Sixteen effect sizes were larger than 3.0 and could be viewed as outlier effect sizes that could have serious impact on the mean effect size and the moderator analyses. Therefore, to avoid an overestimation of the effectiveness (Lipsey and Wilson, 2001), we recoded all effect sizes greater than three to *d* = 3.0, because it seems that those effect sizes are not realistic within intervention studies (Beelmann, personal communication). Next, calculating mean effect sizes, we aggregated the data of multiple dependent variables that measured changes in one dependent construct (outcome cluster). As a result, we obtained 78 effect sizes, with each effect size referring to one dependent construct in one study (*d*_*c,i*_, Level III). Again, we calculated mean effect size, to synthesize the effect sizes of the dependent constructs into one mean effect size per study (*d*_*i*_, Level II). Then, we planned to analyze data across studies (Level I) using methods developed by Hedges and Olkin (1985). Because studies with a greater sample size are generally more conclusive than smaller studies, each study was weighted with:

$$\begin{array}{l} \omega_{i}=\;\frac{2\left(N_{i}\right)N_{iIG\;}N_{iCG\;}}{2{\left(N_{i}\right)}^{2}+N_{iIG\;}N_{iCG\;}d_{i}} \end{array}$$

To get one overall weighted mean effect size, we calculated:

$$\begin{array}{l} d_{MEAN}=\;\frac{\sum_{i=1}^{k}d_{i}\omega_{i}}{\sum_{i=1}^{k}\omega_{i}} \end{array}$$

To obtain weighted mean effect sizes for outcome clusters (dependent constructs), we used the same formula replacing the mean effect sizes per study (*d*_*i*_) with the mean effect sizes per construct per cluster (*d*_*c,i*_, Level III). Our analysis did not correct for multiple testing in order not to lose power. Instead, the chosen method is strong in avoiding Type I error. Having conducted 78 tests, it is important to note that four results may have been randomly significant (expecting five randomly significant results out of 100 tests).

### Assessment of Heterogeneity

Heterogeneity is an important issue in meta-analysis. It deals with the question of whether all included studies measure the same intervention effect. Usually, if there is a more general research question and there are less strict inclusion criteria, as it is the case in our analysis, we expect that we can observe more differences between studies in content- and method-related characteristics. We analyzed the heterogeneity of the effects using *Q*-statistics. A significant *Q* means that the heterogeneity is larger than we would expect from sampling error. In this case, we would need to calculate a random instead of a fixed-effect model (Lipsey and Wilson, 2001). In a random-effect model, we use a new inverse variance component resulting in greater confidence intervals of the effect sizes. From *Q*-statistics, we can calculate *I*-square, which is an estimator for the inconsistency of the results (Higgins et al., 2003). It is interpreted as the percentage of variance of the effect that is caused by heterogeneity (25% = low heterogeneity, 50% = moderate heterogeneity, 75% = high heterogeneity; Cohen, 1988).

### Analysis of Outliers and Publication Bias

Due to the file-drawer problem (i.e., the problem that studies failing to produce a statistically significant result are less likely to be published than those that do produce a statistically significant result), meta-analyses are in danger of overestimating the effects of an intervention (Rosenthal, 1979). We used a funnel plot and trim-and-fill analysis, two methods for assessing publication biases, to explore whether this might be the case in our analysis. We also investigated outliers that might bias the results.

### Assessment of Sensitivity

To address issues of heterogeneity and to obtain a better understanding of which study characteristics might influence the assessment of effect sizes, we conducted sensitivity analysis for categorical variables (METAF, Macros from David B. Wilson; Lipsey and Wilson, 2001) and for metric variables (METAREG, Macros from David B. Wilson; Lipsey and Wilson, 2001). The study characteristics were incorporated as moderators of the effects.

To compare different control group types (e.g., waiting-list control group, physical exercise control groups), we additionally conducted separate analysis of studies with more than one control group, to ensure that all other study characteristics remained constant.

### Analysis of Follow-Up Data

To obtain information about long-term effects, we created a separate file to analyze follow-up data. Using the same procedure as described above, we calculated the effects from pre-test to follow-up values.

## Results

### Study Characteristics

#### Countries of Publication

**Figure 3:**
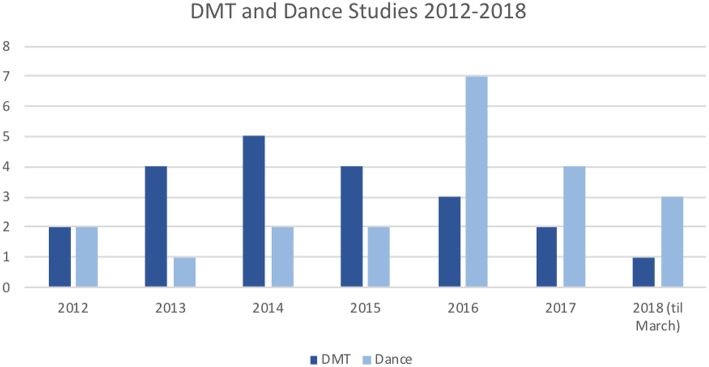
Included studies per year with DMT or dance intervention.

The incorporated studies came from 14 different countries. Most studies were conducted in Germany (11 studies, 26.8%). Overall, 25 studies (60.98%) were conducted in Europe. In addition, eight studies (19.51%) were conducted in Asia, three studies (7.32%) in North America, two studies (4.88%) in Australia, two studies in South America (4.88%), and one study in Africa (2.44%). Generally, one can observe that most studies stemmed from “Western” countries (Germany, UK, USA, Canada, Australia, Mediterranean, and Scandinavian countries). Most Asian studies were conducted in Hong Kong, Korea, and Japan. The remaining studies stemmed from Malaysia, India, and Nigeria. We found seven studies that had not been published yet (Aweto et al., 2012; Ventura et al., 2016; De Natale et al., 2017; Koch, 2017; Pohlmann et al., 2017; Abraham et al., 2018; Aithal and Karkou, 2018). Figure 3 displays the number of studies that met the inclusion criteria found per year. The search dates were January 2012 to March 2018. While dance and DMT studies were equal in numbers in 2012, there were more DMT studies in 2013–2015, and more dance studies in 2016–2018.

#### Samples

Twenty-nine studies (70.73%) implemented the intervention in a clinical sample, and 11 (27.5%) were conducted with a non-clinical (educational) sample. From the clinical populations, 11 were diagnosed with affective disorders (depression), or reported feelings of stress, sadness, or anxiety. Six samples were diagnosed with Parkinson's disease, five with schizophrenia, four with ASD, and two with cognitive impairments. Overall, there were more female than male participants (percentage female participants: M = 65.66%, SD = 27.65). Comparing intervention and control group, the distribution of female and male participants was even.

#### Interventions

About half of the studies were DMT intervention studies (21 studies); the other half were dance intervention studies (20 studies). In the *DMT group*, 16 interventions were provided by a trained dance movement therapist, at least four of them were also qualified clinical psychologists. In four trials, descriptions of qualification of therapists were missing or dance movement therapists in training (Aweto et al., 2012; Serrano-Guzmán et al., 2016; Guerra-Balic et al., 2017; Wiedenhofer and Koch, 2017). We allocated them to the DMT group because the intervention was predominantly DMT (distinguishing criterion is described above). In the study by Abraham et al. (2018), classification of qualification of therapist was difficult. The intervention was provided by a physiotherapist specialized at “dynamic neuro-imagery” intervention, a DMT-like introspection intervention. We decided to allocate the study to the DMT group, because of content-related similarities of the intervention to DMT. In the *dance intervention group*, seven interventions were conducted by an instructor with dance education. Additionally, seven interventions were conducted by exercise instructors with other backgrounds (physical education, physiotherapy, nursing). Six trials contained no description about qualification of dance instructors and were allocated to the cluster because of content-related similarities to the other dance intervention studies.

The *DMT studies* differed in the technique, e.g., the technique by Marian Chace (“Chacian approach”, a standard method in DMT; Sandel et al., 1993), dance therapy with ethno-elements, DMT modified to suit Chinese culture, manualized body psychotherapy (conducted by dance therapists; manual of Röhricht and Papadopolous, unpublished), and dance therapy with elements of creative movement play (for more extensive description of intervention methods in DMT, see Koch, 2019). Dance interventions used various dance styles, mostly traditional folk dance or cultural dance forms (Irish set dancing, Greek traditional dance, Flamenco, Poco-Poco dance, Belly dance, Tango), further couple dance (Latin dance), and contemporary dance (jazz, modern, creative dance). All interventions took place in groups. DMT and dance interventions were modified to the age of the participants. In the studies of Meric and Ilhan (2016) and Panagiotopoulou (2018), the researcher and the implementing person were identical. The quality of intervention description differed widely across studies. Fifteen percent of the studies described the intervention very rudimentarily, 22.5% moderately, 22.5% more detailed, and 40% replicable, which is a clear increase in replicable descriptions compared to the seven studies in the former meta-analysis by Koch et al. (2014).

#### Control Groups

To control for unspecific effects, about one third of the studies (31.7%) used active control groups [e.g., listening to music, cycling, Pilates, physical education/sports exercises (physical exercises), psychoeducation, meditation, relaxation exercises], and 65.9% of the studies used a passive control group (e.g., waiting-list group or treatment as usual). One control group description was missing (Meric and Ilhan, 2016).

#### Duration of Interventions

Most of the included studies were implementing the intervention over a longer period of time (Mean = 11.41 weeks, SD = 7.89, Min = 1 week, Max = 32 weeks). Only the studies by Campion and Levita (2014) and Wiedenhofer and Koch (2017) implemented one single-dance/DMT session and assessed short time effects immediately after the intervention. Their sessions lasted for 5 min in the study by Campion and Levita (2014) and for 50 min in the study by Wiedenhofer and Koch (2017). The other sessions were usually carried out two times per week (Mean = 1.85, SD = 1.05, Min = once per week, Max = 4 times per week), and lasted for 1 h or 1 1/2 h (Mean = 71.32 min, SD = 19.02, Min = 40 min, Max = 120 min).

#### Statistical Tests and Assessment Methods

To assess and compare pre–post differences of the dependent variable in the intervention and control group, either *t*-tests, ANOVAs, or MANOVAs were calculated. Mostly self-report questionnaires were used for assessment (49%), followed by observation rating scales (17.6%), cognitive tests (13.4%), tests of motor functioning (11.1%), interviews (6.5%), and psychophysiological measures (2.3%).

#### Sample Sizes and Randomization

The meta-analysis included studies with large and very small sample sizes (*N:* Mean = 57.90, SD = 49.61, Min = 11, Max = 275). In 21.95% of the cases, the samples were smaller than *N* = 30, 34.15% were *N* = 30–50, 31.70% *N* = 50–100, and 12.20% were larger than *N* = 100. The studies with the largest sample sizes are contributing most to the results (weighted mean effect sizes); these are (Priebe et al., 2016) (*N* = 275), (Vankova et al., 2014) (*N* = 162), (Bräuninger, 2012a) (*N* = 162), (Ho et al., 2016a) (*N* = 139), and (Duberg et al., 2013) (*N* = 101). In general, smaller studies are less conclusive and generalizable than studies with larger sample sizes (unless their methodological quality is significantly better). Therefore, we weighted their results discriminatingly, calculating weighted mean effect sizes (see previous paragraph). Notably, most studies did not meet established criteria or rules of thumb that would define desirable sample sizes to conclude effects for a certain population (Harris, 1985; Kraemer and Thiemann, 1987; Green, 1991). Nevertheless, it is assumed that (apart from publication bias) studies with a small sample size would not significantly bias the results of meta-analyses but contribute important information, for example, about heterogeneity and the effects in sub-groups (Higgins et al., 2003; Grainge, 2015).

Randomization is an important criterion for the reliability and validity of estimated effect sizes, because it addresses the issue of comparability of the inspected groups. Fifty-two and a half percent (52.5%) of the studies used randomization as group allocation process, 22.5% used quasi-randomization techniques, and 25% of the studies used no randomization techniques (group allocation was based on self-selection or purposive sampling). Of the 10 non-randomized studies, only 5 included extra assessments of baseline differences, showing that there were significant differences in three of them (Pylvänäinen et al., 2015; Adam et al., 2016; Aithal and Karkou, 2018). The inclusion of studies with baseline differences is justified here, because our methodological approach takes those differences into account when calculating effect sizes. The studies from Biber (2016), Guerra-Balic et al. (2017), Meric and Ilhan (2016), and Panagiotopoulou (2018) used no statistical tests to assess baseline differences.

#### Dropouts

Thirty-one of the 41 studies reported dropout rates (Mean = 13.55%, SD = 15.18, Min = 0%, Max = 57.38%). Analysis of dropout rates revealed that 7 of the 31 studies reported a dropout that is higher than 30% (Pinniger et al., 2012, 2013; Röhricht et al., 2013; and Rios Romenets et al., 2015; Hildebrandt et al., 2016; Martin et al., 2016; Pohlmann et al., 2017). This was in many cases due to the fact that severely impaired patients with schizophrenia or depression had to actively travel to outpatient treatment, requiring a strong motivational state, which is precisely one of the problems in these disorders.

#### Overall Quality of Included Studies

Because we applied mild selection criteria, the methodological quality of the included primary studies is considerably heterogeneous, and risk of bias is a concern in most of the included studies. Referring to the Cochrane Collaboration's tool for assessing risk of bias in randomized trials, there are six domains of bias that should be considered: selection bias, performance bias, detection bias, attrition bias, reporting bias, and other sources of bias (Higgins et al., 2011). Selection bias relates to the group allocation of participants. As reported above, a quarter of the studies had a great risk of selection bias (or in this respect), because no randomization or quasi-randomization tools were used. Additionally, in 12.2% of the trials, no baseline characteristics were checked. Secondly, performance bias relates to blinding of the participants and staff involved. Any of the investigated trials might be affected by this risk of bias (Rosenthal effect), because blinding is a challenge in any type of intervention study. While it is possible to conceal, which is the experimental vs. control condition in active control group trials, it is more difficult in waiting list designs, such as is the case in many of the DMT and dance interventions here. However, it is plausible that self-report measures and external rating scales are more affected by performance bias than cognitive or motor tests or physiological data. Detection bias addresses blinding of the researcher, who assesses and analyses the results. We were not able to extract this information from most of our primary trials, but we assume that researchers usually know the treatment allocation of the participants. Attrition bias is about the issue of the amount and handling of incomplete outcome data. As we assessed dropout rates (see section above), we can state that about 42% of the studies are in danger for this domain of risk of bias. Because selective reporting (reporting bias) is hard to extract from primary trails, we did not assess this domain. Concerning other risks of bias (e.g., conflicts of interest), in two studies, the researcher was also the implementing person (Meric and Ilhan, 2016; Panagiotopoulou, 2018). To conduct further analyses, we used dichotomous coding (higher-risk vs. lower-risk studies), including all studies in the higher-risk category that had a total *N* smaller than 30 or which yielded issues in any of the assessed domains of risk of bias (group allocation, attrition bias, other bias). A more detailed description on study characteristics is provided in Table 1 (study characteristics chart).

### Overall Effect

Overall, we synthesized data from 2,374 participants from 41 studies (more than twice the number of the last general meta-analysis by Koch et al., 2014). According to Cohen (1988), effect sizes between *d* = 0.2 and *d* = 0.5 are small effects, effect sizes between *d* = 0.5 and *d* = 0.8 are medium effects, and effect sizes larger than *d* = 0.8 are large effects. The mean effect sizes per study (Level II, *k* = 41) varied between one small negative effect size and large positive effect sizes (Unweighted Mean: *d* = 0.67, Min: *d* = −0.17, adjusted Max: *d* = 2.96). The maximum effect size is not bigger than 3.0, because of the adjustments we obtained at Level III. The unadjusted Maximum would be *d* = 5.07. Further details about the distribution of effect sizes across studies are described in the paragraphs below.

We calculated a fixed-effect model to obtain a weighted mean effect size across studies (Level I). The weighted overall fixed effect was *d* = 0.48 (*p* < 0.001, CI_min_ = 0.40, CI_max_ = 0.57). The analysis of heterogeneity showed a high inconsistency of results (*Q* = 127.52, *df* = 40, *p* < 0.001, *I*^2^ = 72.62%). Therefore, we calculated a random-effect model as recommended in Lipsey and Wilson (2001). The estimated weighted overall random effect was *d* = 0.60 (*p* < 0.001, CI_min_ = 0.44, CI_max_ = 0.76). In the following paragraphs, we will only report results that were calculated with the random-effect model.

### Effect Sizes According to Outcome Cluster

The weighted mean effects sorted by constructs (outcome clusters) are summarized in the chart below. The analysis yielded a significant effect for each outcome cluster. The effect for interpersonal skills was the largest one, followed by quality of life and (psycho-)motor skills. Furthermore, all effects but the effect for (psycho-)motor skills showed high heterogeneity. Since we calculated a random-effect model, all effects showed broad confidence intervals (see Table 2).

**Table 2:** Effect sizes according to outcome cluster.

**Construct**	***k***	**Mean ES, d_c_**	**CI**	**SE**	***p***	***Q***	***p***	***I*^2^ %**
Quality of life	20	0.67^***^	0.41–0.99	0.133	<0.001	89.30	<0.001	78.72
Affect	23	0.56^***^	0.34–0.79	0.115	<0.001	88.65	<0.001	75.18
Interpersonal skills	9	0.85^***^	0.41–1.28	0.222	<0.001	38.61	<0.001	78.15
Cognitive skills	10	0.53^**^	0.13–0.93	0.204	0.009	28.51	0.001	68.43
(Psycho-)motor skills	10	0.65^***^	0.36–0.96	0.152	<0.001	14.61	0.102	38.39
Residual	6	0.47^*^	0.06–0.88	0.208	0.025	20.04	0.001	75.05

k, number of studies; ES, effect size; CI, confidence interval; SE, sampling error;

* p < 0.05;

** p < 0.01;

*** *p < 0.001; Q, parameter of heterogeneity. The first p-value on the left refers to the mean effect size, whereas the p-value on the right side refers to Q*.

### Subanalysis

The subanalysis of anxiety and depression revealed that the differentiation of the two constructs did not lead to more homogeneous results. Depression yielded a slightly larger effect than anxiety. Both effects were medium and showed high heterogeneity. The effect for physiological variables was large but heterogeneous; the effect for positive symptoms in schizophrenia reached significance on the *p* < 0.1 level and was also heterogeneous (see Table 3).

**Table 3:** Effect sizes according to sub-clusters.

**Construct**	***k***	**Mean ES, d_c_**	**CI**	**SE**	***p***	***Q***	***p***	***I*^2^ %**
Anxiety	9	0.47^**^	0.09–0.84	0.192	0.015	42.88	<0.001	81.34
Depression	18	0.54^***^	0.30–0.78	0.124	<0.001	65.35	<0.001	73.99
Physiological variables	2	0.88^**^	0.22–10.54	0.338	0.009	2.63	0.105	61.98
Schizophrenia (pos symptoms)	4	0.40^*^	−0.01–0.79	0.205	0.05	7.99	0.046	62.45

k, number of studies; ES, effect size; CI, confidence interval; SE, sampling error;

* p < 0.05;

** p < 0.01;

*** *p < 0.001; Q, parameter of heterogeneity*.

### Analysis of Outliers and Publication Bias

The one-session study from Campion and Levita (2014) was the only one to show a (small) negative effect (*d* = −14). Other studies that were at the lower end of the distribution were Koch et al. (2015a,b) that included an anti-violence training (*d* = 0.02) and Ho et al. (2016a) that aimed to improve the quality of life of cancer patients (*d* = 0.06). On the other side of the distribution, we could observe large effect sizes (*d* = 1.8 up to *d* = 2.96) in the studies of Cruz-Ferreira et al. (2015), Biber (2016), Teixeira-Machado et al. (2017), and Aithal and Karkou (2018). If we had not recoded the effect sizes from Chiang et al. (2016), Pohlmann et al. (2017), Teixeira-Machado et al. (2017), and Aithal and Karkou (2018) at Level IV, they would have been even larger (up to *d* = 5.07). Possible reasons for the effect size distribution across studies are detailed in the next paragraphs.

**Figure 4:**
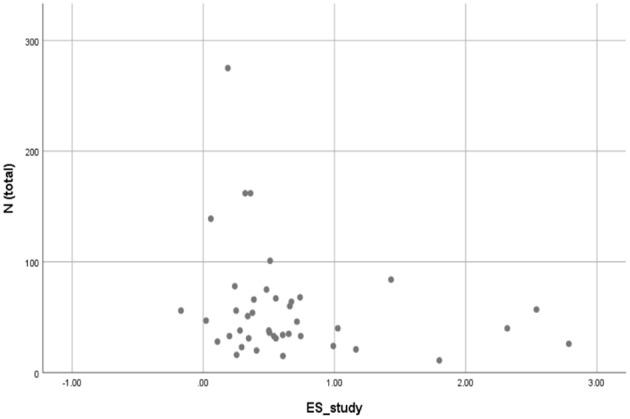
Funnel plot.

To analyze the distribution of effect sizes and to address the issue of publication bias, we created a funnel plot (see Figure 4) that shows the distribution of unweighted effect sizes as a function of sample sizes. The distribution would be asymmetrical in case of a publication bias, because then small sample studies with positive effect sizes would be published, whereas small sample studies with no positive effects would remain unpublished (Lipsey and Wilson, 2001).

The diagram in Figure 4 shows a skewed distribution to the right; that is, the large positive effect sizes on the right have no equivalent on the left. This is an indication of a publication bias. Therefore, we conducted a regression analysis (independent variable: sample size, dependent variable: unweighted effect size). There was a small negative gradient parameter that did not reach significance (β = −0.003, *p* = 0.147). We can thus assume that the publication bias does not have a great impact on the results. Nevertheless, the fact that the gradient parameter did not reach significance does not mean that there is no publication bias at all. We also conducted trim-and-fill analysis as a more elaborate method to explore publication bias using the random-effect model and an *L*_0_ estimator (Duval and Tweedie, 2000). The number of estimated missing studies on the left side, meaning the number of studies that are assumed not to be found due to publication bias, was zero. Thus, the new estimated overall effect did not differ from the original one.

### Assessment of Sensitivity

As described above, we conducted analysis of sensitivity using study characteristics as metric and categorical moderator of the effect sizes per study (*d*_*i*_).

### Metric Variables

Regression analyses revealed that no metric variable alone reached significance as a moderator of effect sizes (see Table 4 below). *N* total was closest to significance, indicating that studies with a greater sample size yielded smaller effects. This can be interpreted as a result of the file-drawer problem, risk of bias of smaller studies, or as an estimator of quality of implementation (we can assume that, in smaller samples, the intervention was implemented more appropriate to the individual) (see Table 4).

**Table 4:** Metric moderators.

**Moderator**	**β**	***p***
*N* total	−0.0025	0.074
Percentage women (total)	0.1739	0.592
Relation women EG vs. CG	0.2861	0.477
Length process (weeks)	0.0096	0.356
Length session (min)	−0.0024	0.545
Frequency (times per week)	0.1375	0.091
Dropout percentage EG	−0.0087	0.257
Dropout percentage (total)	−0.0092	0.172

*EG, experimental group; CG, control group; β, gradient parameter; p, significance parameter*.

Overall, 40.84% of variance (*R*^2^) was explained by metric moderators.

### Categorical Variables

We conducted sensitivity analysis with the following categorical moderators: country, publication status, higher vs. lower risk of bias, age range, clinical vs. non-clinical sample, diagnosis, DMT vs. dance intervention, qualification of implementing person, quality of intervention description, one-session vs. process, randomization, dropout rates, type of control group activity, and measurement type.

Two moderators were significant on a *p* < 0.05 level: DMT vs. dance intervention studies (between variance: *Q* = 5.54, *df* = 1, *p* = 0.019), and qualification of implementing person (between variance: *Q* = 8.89, *df* = 3, *p* = 0.031). Trials with DMT interventions yielded significant but slightly lower effect sizes (*d* = 0.35, *p* < 0.001) than trials with dance interventions (*d* = 0.81, *p* < 0.001). In addition, the results of the group with DMT interventions were consistent (*I*^2^ = 3.47), whereas the results in the dance intervention group were inconsistent (*I*^2^ = 77.96). Country, higher vs. lower risk of bias, and age range were significant moderators of effect sizes on a *p* < 0.1 level. In children, teenagers, and seniors, larger effect sizes were observed than in adults, but the effect sizes in adults were more consistent. Furthermore, lower-risk studies yielded smaller effect sizes than higher-risk studies and were more homogeneous. In clinical trials, effect sizes were smaller than in non-clinical trials, but the results remained more consistent than in non-clinical trials (weighted mean effect sizes sorted by group, see Table 5).

**Table 5:** Categorical moderators.

**Variable**	**Between variance** **(*Q*, *df*, *p*)**	**Group 1 (*d*, *p*, *I*^2^)**	**Group 2 (*d*, *p*, *I*^2^)**	**Group 3 (*d*, *p*, *I*^2^)**	**Group 4 (*d*, *p*, *I*^2^)**	**Group 5 (*d*, *p*, *I*^2^)**	**Group 6 (*d*, *p*, *I*^2^)**
Country	9.68 5 0.085	Germany (0.46, 0.002, <1%)	North/Middle/West Europe (0.44, 0.028, <1%)	South/East Europe (0.81, <0.001, 61.26%)	Far East (0.37, 0.142, <1%)	USA/Australia/Canada (0.45, 0.065, 20%)	Others (1.17, <0.001, 61.79%)
Higher vs. lower risk of bias	3.27 1 0.071	Lower risk (0.48, 0.000, 8.68%)	Higher risk (0.76, 0.000, 34.48%)	–	–	–	–
Randomization	2.27 2 0.320	No randomization (0.85, <0.001, 44.17%)	Quasi- randomization (0.48, 0.003, <1%)	Randomization (0.57, <0.001, 37.48%)	–	–	–
Age range	10.71 5 0.098	Children (1.40, <0.001, 79.51%)	Teenager (0.93, 0.003, 79.29%)	Young adults (0.55, 0.051, 27.18%)	Younger + older adults (0.46, 0.000, <1%)	Older adults (0.30, 0.172, <1%)	Seniors (0.83, <0.001, 43.75%)
Diagnoses	4.17 5 0.654	Cognitive impairment (0.87, 0.017, 68.75%)	Affective disorders/stress (0.57, <0.001, <1%)	Developmental disorders (0.41, 0.106, <1%)	Schizophrenia (0.40, 0.075, <1%)	Parkinson (0.53, 0.033, <1%)	None (0.90, <0.001, 72.41%)

*df, degree of freedom (df = k – 1)*.

### Control Group Type

Across all studies, the type of control group activity was not a significant moderator of effect sizes. A different picture resulted, when we only included studies with more than one control group into the analysis (*k* = 9, active control groups: meditation, physical exercises). For these studies, the weighted mean effect size remained significant when compared to non-active control groups (in most cases, waiting list CGs), but declined to a visible but non-significant effect when compared to active control groups (*d* = 0.43, *p* = 0.009 vs. *d* = 0.21, *p* = 0.126). Especially when compared to meditation intervention, the effects declined to almost zero, which means that the two interventions are equally effective.

### Measurement Type

Type of measurement was a significant moderator of effect sizes (between variance: *Q* = 97.92, *df* = 5, *p* < 0.001). Observation rating scales yielded the largest effect sizes (*d* = 1.34, *p* < 0.001, *I*^2^ = 76.01%). They were followed by physiological data (*d* = 0.71, *p* = 0.001, *I*^2^ = n.s.), motor tests (*d* = 0.58, *p* < 0.001, *I*^2^ = n.s.), self-report-questionnaires (*d* = 0.42, *p* < 0.001, *I*^2^ = n.s.), cognitive tests (*d* = 0.30, *p* = 0.002, *I*^2^ = n.s.), and interviews (*d* = 0.25, *p* = 0.001, *I*^2^ = n.s.). We thus can assume that the type of measurement, especially observation rating scales, is one of the most significant sources of heterogeneity across studies. We also checked whether observation rating scales were more frequently used in dance intervention studies than in DMT studies, which was not the case.

### Explorative Analyses

Since sensitivity analysis revealed type of intervention as a significant moderator, we decided to exploratively conduct separate analyses of the DMT and dance intervention trials (the results are reported in the Tables 6, 7 below; they were also reported briefly in the paragraph above).

**Table 6:** Effect sizes only in DMT studies.

**Construct**	***k***	**Mean ES**	**CI**	**SE**	***p***	***Q***	***p***	***I*^2^ %**
Overall	21	0.35^***^	0.23–0.46	0.059	<0.001	20.72	0.414	3.47
Quality of life	10	0.32^*^	0.02–0.63	0.155	0.036	1.50	0.997	n.s.
Affect	12	0.51^**^	0.18–0.85	0.171	0.003	9.49	0.577	n.s.
Interpersonal skills	6	0.49	0.00–0.97	0.249	0.051	0.43	0.994	n.s.
Cognitive skills	3	0.26*	(−0.48)−0.99	0.268	0.011	0.23	0.890	n.s.
Motor skills	2	0.30	(−0.28)−0.88	0.297	0.315	0.14	0.712	n.s.

k, number of studies; ES, effect size; CI, confidence interval; SE, sampling error;

* p < 0.05;

** p < 0.01;

*** *p < 0.001; Q, parameter of heterogeneity*.

**Table 7:** Effect sizes only in dance intervention studies.

**Construct**	***k***	**Mean ES**	**CI**	**SE**	***p***	***Q***	***p***	***I*^2^ %**
Overall	21	0.81^***^	0.52–10.11	0.149	<0.001	86.22	<0.001	77.96
Quality of life	10	1.02^***^	0.69–1.34	0.165	<0.001	23.41	0.005	61.55
Affect	11	0.69^***^	0.34–1.04	0.177	<0.001	16.63	0.083	39.86
Interpersonal skills	3	1.65^***^	0.90–2.40	0.383	<0.001	8.71	0.013	65.57
Cognitive skills	7	0.68^**^	0.16–1.21	0.268	<0.001	10.85	0.093	44.65
Motor skills	8	0.76^***^	0.44–1.08	0.163	<0.001	8.67	0.277	19.23

k, number of studies; ES, effect size; CI, confidence interval; SE, sampling error;

* p < 0.05;

** p < 0.01;

*** *p < 0.001; Q, parameter of heterogeneity*.

We also analyzed weighted mean effect sizes according to outcome clusters separately for the two groups. For quality of life, the effect remained significant in both clusters, but showed consistency only in the DMT group. For clinical outcomes, both effects were significant but heterogeneous. Related to interpersonal skills, there was a significant but inconsistent effect in the dance intervention group and a significant (*p* = 0.05) and homogeneous effect in the DMT group. In the cognitive skills cluster, the effect remained significant but heterogeneous in the dance intervention group, but did not reach significance in the DMT group (*k* = 3 trials). The effect for motor skills was significant and consistent in the dance intervention group; in the DMT group, it was not significant (*k* = 2 trials).

In Tables 6, 7, changes in depression and anxiety are the main outcomes under *affect changes*. It is notable that the *changes in interpersonal skills* just barely missed significance in DMT studies. Considering the high heterogeneity of outcome measures in this domain, they need in any case to be further investigated (plus more standardized outcome measured need to be developed). *Changes in motor skills* are most pronounced and consistent in dance intervention studies, with the most and the most rigorous evidence from studies of interventions for Parkinson patients (e.g., Hackney and Bennett, 2014; Sharp and Hewitt, 2014; Loetzke et al., 2015). Changes in motor skills are usually not in the focus of DMT studies. The non-significant results on motor skills confirm DMT as a psychotherapeutic intervention.

### Assessment of Follow-Up Data

In order to obtain information about the long-term effects of DMT and dance interventions, we analyzed the available follow-up data (see Table 8). Eight studies reported follow-up data, the mean period from post- to follow-up assessment was 22 weeks. In most studies, the effect remained constant. The largest decline was observed in the study by Bräuninger (2012a,b). The effect was reduced by half, but remained significant. In Baptista et al. (2012), Pylvänäinen et al. (2015) and Priebe et al. (2016), there was a slight decline, which was close to zero; in De Natale et al. (2017), there was a slight increase. In the studies from Pinniger et al. (2012), Duberg et al. (2013), and Cruz-Ferreira et al. (2015), there was a large increase (e.g., in Pinniger the effect tripled). The authors explain this unusual increase with an increase in mindfulness (detachment from negative thoughts, ruminations, and worries, which led to a reduction in symptom severity; Ree and Craigie, 2007) and a personal bond between the participants as a learning process, which became more effective after the participants had practiced for a while.

**Table 8:** Effect sizes follow-up.

**Study**	**Period follow-up (weeks)**	**Pre–post effect **(**d_i_)**	**Pre-follow-up effect **(**d_i_)**	**Post-follow-up effect **(**d_i_)**	**Weight (ω_i_)**
Baptista et al. (2012) -D	32	0.48	0.39	−0.09	18.22
Bräuninger (2012a,b)	24	0.64	0.38	−0.26	37.12
Cruz-Ferreira et al. (2015) -D	24	2.54	3.00	0.46	7.83
De Natale et al. (2017)	8	0.25	0.39	0.14	3.91
Duberg et al. (2013) -D	48	0.51	0.81	0.30	24.40
Pinniger et al. (2012)	4	0.27	0.82	0.55	11.59
Priebe et al. (2016)	24	0.19	0.14	−0.05	68.43
Pylvänäinen et al. (2015)	12	0.74	0.65	−0.09	7.18
Weighted mean (*p*)	22	0.67 (0.001)	0.79 (0.001)	0.05 (0.613)	–

*The bottom line displays the weighted mean effect sizes across the eight studies and a significance parameter (p-value); D, dance intervention study*.

## Discussion

### Summary of Results

In this meta-analysis, we investigated the effectiveness of DMT and dance interventions on health-related psychological outcomes. We included 41 primary trails published between 2012 and March of 2018 that contained a total of 2,374 participants. Twenty-one of these trials considered DMT interventions, whereas 20 trials considered dance interventions. We grouped the dependent variables into six outcome clusters: quality of life (*k* = 20 trials), clinical outcomes (23 trials; sub-analysis: depression, anxiety), interpersonal skills (*k* = 9 trials), cognitive skills (*k* = 10 trials), (psycho-)motor skills (*k* = 10 trials), and residuals (*k* = 6 trials; physiological data, positive symptoms schizophrenia). The overall mean effect size was *d* = 0.60 (*p* < 0.001, CI_min_ = 0.44, CI_max_ = 0.76), which is a significant medium effect (Cohen, 1988). Analysis of heterogeneity yielded that an estimated 71.62% of variance of results can be traced back to dissimilarity of results, which makes the interpretation of results more difficult. In the outcome clusters, we also obtained significant medium effect sizes [quality of life: *d* = 0.67, affect: *d* = 0.56, cognitive skills: *d* = 0.53, (psycho-)motor skills: *d* = 0.65, residuals *d* = 0.47] and one significant large effect size (interpersonal skills: *d* = 0.85). This could be due to the fact that in the interpersonal skills cluster, many trials assessed dependent variables with observation rating scales, which yielded larger effect sizes in general. Sub-analyses revealed that the mean effect in the anxiety cluster was as large as the effect in the depression cluster. Physiological data yielded a mean large, significant effect size, whereas improvements of schizophrenia yielded a mean small effect size, which reached the *p* < 0.1 level. Assessment of heterogeneity in the outcome clusters revealed that all mean effects, except the effect for (psycho-)motor skills, remained inconsistent (i.e., significant *Q, I*^2^ larger 60%). Furthermore, the larger the mean effect, the larger the heterogeneity of results, which indicates that larger effects were produced by outliers rather than by consistently higher effect sizes (see end of the next paragraph).

In order to identify which study characteristics contributed most to dissimilarities of results, we conducted a sensitivity analysis. Most importantly, we found that the type of intervention (DMT vs. dance) was a significant moderator of results. Therefore, it was reasonable to analyze data in two separate groups to obtain more meaningful results:

In the *DMT group*, we obtained smaller but more consistent effects. The *overall medium effect* was small, significant, and consistent/homogeneous (*d* = 0.30, *p* < 0.001, non-significant *Q, I*^2^ = 3.47). For the individual outcomes, the effects varied between *d* = 0.26 and *d* = 0.51 and were all homogeneous. The effects for quality of life, affect, and cognitive skills remained significant, whereas the effect for interpersonal skills reached the *p* < 0.1 level. Effects of motor skills were non-significant, thus confirming DMT as a mainly psychotherapeutic intervention.

In the *dance cluster*, we observed larger but less consistent effects. The *overall medium effect* was large, significant but non-consistent/heterogeneous (*d* = 0.81, *p* < 0.001, significant *Q, I*^2^ = 77.96). The effects in the outcome cluster varied between *d* = 0.68 and *d* = 1.65. They were all significant, but only the effects for (psycho-)motor skills, cognitive skills, and affect were consistent (non-significant *Q, I*^2^ < 50%). Apart from Koch et al. (2015a,b) and Aithal and Karkou (2018), all outliers we mentioned in the “Analysis of outliers and publication bias” section were part of the dance intervention group. The consistent effects for (psycho)motor skills were carried by the dance for Parkinson studies.

Dance studies seem to uphold certain characteristics that produce a broader range of results, especially in the large-positive spectrum of effect sizes (i.e., greater than *d* = 1.0) that are not evident in DMT studies. One factor might be different sample characteristics: Most DMT studies were conducted in a clinical setting, whereas most dance studies were conducted in a non-clinical setting. In severely impaired patient samples, such as in most of the DMT primary studies that entered into this analysis, effects are usually smaller than in non-clinical or subclinical populations (we know this, for example, from pretests of designs with student populations; e.g., Koch, 2011). This may be a major reason why the dance intervention studies have yielded larger medium effect sizes. Non-clinical samples also contained a broader age range. Additionally, in dance interventions, the implementation and methods were more heterogeneous than in the DMT group. In terms of culture, most DMT studies were conducted in Germany and other Western European countries, whereas the majority of dance intervention studies were conducted in non-Western countries. Dance intervention studies tended to have smaller sample sizes, less randomization, and more missing information in the reporting of results.

In sum, we obtained encouraging results, which indicated that DMT and dance have positive effects on various health-related outcomes. Most studies found evidence on the effectiveness of DMT on clinical outcomes (*k* = 12 trials), followed by quality of life (*k* = 10 trials) and cognitive skills (*k* = 3 trials). There was also a tendency that DMT improved interpersonal skills (*k* = 6 trials; *p* = 0.051). We did not find enough studies on (psycho-)motor functioning, physiological changes, and positive symptoms of schizophrenia in the DMT cluster to draw conclusions. Dance interventions improved (psycho-)motor skills (*k* = 8 trials), clinical outcomes (*k* = 11 trials), and cognitive skills (*k* = 7 trials). The high variety of results, especially in the dance cluster, needs more investigation in future studies.

### Specific Research Issues and Practical Implications for Researchers

In our meta-analysis, we also assessed and analyzed various study characteristics to deepen our understanding of factors that influenced the effects of DMT and dance on health-related outcomes. One important issue in most intervention studies is the question about *unspecific and specific effects of the intervention*. Often the fact that we pay attention to the participants already can change their symptoms (Hawthorne effect: firstly described in Roethlisberger and Dickson (1964), and reviewed by McCambridge et al., 2014). Using alternative interventions in the control groups helps to distinguish attention effects from specific effects. Therefore, we assessed control group activity. In the primary trials, one third of the DMT and dance intervention groups were compared to active control groups. The participants followed the following tasks: listening to music, cycling, Pilates, physical exercises, psychoeducation, meditation, or relaxation exercises. Control group activity was not a significant moderator of effect, which means that the effects of DMT and dance interventions were still evident when we controlled for attention effects. In other words, this indicates that there are specific effects of DMT and dance interventions. Control group activity seemed to matter, when we kept all other study characteristics constant, which was only possible in studies with an active and a non-active control group (*k* = 9).

We compared the intervention groups to control groups that participated in physical exercises and meditation exercises. The effects of DMT and dance interventions declined but remained significant. The advantages of DMT and dance interventions were larger compared to physical exercises than to meditation. This indicates that the change mechanisms of DMT and dance possibly lie beyond the pure execution of sports or mindfulness. This is in line with other research on the mechanisms of change in DMT and dance (Bräuninger, 2014; Koch, 2017). The effect sizes observed in DMT and dance intervention groups were about as large as the effect sizes in meditation interventions. There is plenty of evidence that meditation interventions, for example, training programs in mindfulness-based stress reduction (MBSR; Teasdale et al., 2000; Michalak et al., 2008), are successful in treating several psychological conditions (e.g., depression, anxiety, stress, obsessive–compulsive disorders; Bohlmeijer et al., 2010; Fjorback et al., 2011). On basis of this evidence, there is also an increase in promotion and support of mindfulness-based interventions by the health insurance companies, such as free MBSR training programs at work. Thus, observing that DMT and dance interventions seem to be as effective as meditation suggests promoting these kinds of interventions as well. One interesting research issue that we should investigate in more detail is: *What do DMT/dance interventions and mindfulness-based interventions have in common?* Mindfulness is an important component of DMT. For example, mindful investigation of body sensations is part of DMT, with similar techniques as the body-scan exercise in MBSR training programs (Dreeben et al., 2013). Besides that, getting mindfully in contact with other people (therapist or other group members) is an important mechanism of change in DMT. Concerning dance interventions, there is less explicit use of mindfulness, but if we look closer, similar mechanisms of change stand out. One central component frequently mentioned in the literature on mindfulness is the termination of rumination and automatic negative thoughts, which occurs, because there is high concentration, focus on the here-and-now, and a state of mind, which allows the participant to observe feelings and thoughts from a more distant perspective (non-judgmental state of mind). Future research should investigate to what extent these mechanisms are relevant in DMT and dance intervention and how they influence the outcomes of the interventions. Moreover, we assume that an important overarching therapeutic factor in both mindfulness practice and DMT could be introspection (Price and Smith-DiJulio, 2016). Another central mechanism of change in dance and DMT might be the experience of flow (Csikszentmihalyi and Csikszentmihalyi, 1975). It is based on the same principles as mindfulness, such as high concentration, absorption, focus on the here-and-now, physical presence, and joy (Csikszentmihalyi and Csikszentmihalyi, 1975).

In order to find out which populations DMT and dance interventions are indicated for, we investigated the influence of *sample characteristics* on the effect sizes. Age range and clinical vs. non-clinical populations were two correlating moderators on a *p* < 0.1 level. We observed larger mean effects in children and elderly than in younger and older adults. At the same time, there was higher heterogeneity of results in the group of children and elderly. The fact that outcome variables in children and elderly were assessed with observation rating scales more often might contribute to these differences. Furthermore, the larger effects in children may also have come about because there were more prevention studies or studies in educational context with children and older adults, whereas the adults were mostly severely impaired clinical populations. Prevention studies yielded larger effects but higher heterogeneity than clinical studies. Again, this means that rather than more pronounced effects, there are outliers at the large-positive spectrum of effect sizes. From these results, we cannot come to recommendations about the indication of treatment in certain populations. Further research is needed to ascertain moderators that produce the variety of results. Regarding *outcomes characteristics*, DMT is indicated when a psychological change is intended, whereas dance interventions are indicated to improve motor skills (the consistent effect only occurred on those).

The so-called *method effect* (“any characteristic of measurement procedure contributes to variance of scores”; Maul, 2013) is a widely discussed limitation of intervention studies. Thus, we assessed and analyzed methodological characteristics of studies. We found one significant moderator, *type of measurement*, and two moderators that reached a *p* < 0.1 level, *country of publication* and *methodological quality of study* (risk of bias, randomization, sample size). Observation rating scales yielded the largest effect sizes followed by physiological data, motor tests, self-report questionnaires, cognitive tests, and interviews. Some measurements may be superior to detect changes or more sensitive to certain risks of bias, therefore producing larger effects than others. For example, observation rating scales and self-report questionnaires are more likely to be affected by expectation effects, which will be discussed further in the limitations of the present study paragraph. However, it is important to mention that method effects can also systematically bias the results. There was also a correlation between country of publication and methodological characteristics of the trials (risk of bias, randomization, sample size), with both factors having a slight influence on the observed effects. This is not surprising, because standards and traditions of research vary between countries. In countries with less evidence-based research traditions, methodological standards tend to be less strict and resulting effects tend to be larger. However, the effect of country could also be related to other cultural factors such as experience with the specific dance form, or the (sub-)cultural value system around dance, which may all influence motivation for and responsiveness to treatment.

We also analyzed whether the *intensity of treatment* had an influence on effect sizes. The intensity of treatment was indicated by three factors: duration of the whole treatment, duration of one session, and frequency of treatment. None of these factors reached significance. Frequency of treatment was closest to significance (β = 0.1375, *p* = 0.091), indicating that higher frequency of treatment corresponded with slightly higher effect sizes.

In the last paragraphs, we discussed moderators that showed a significant influence on the effects of DMT and dance in our sensitivity analysis. However, we assume that there are *more potentially important moderators of effects*, which we were not able to address in our analysis. In the literature, there are factors that we can allocate to four clusters: (a) factors that relate to characteristics of the participants, (b) factors that relate to characteristics of the intervention, (c) factors that relate to characteristics of the implementing person, and (d) factors of environment and factors that relate to the relation between persons involved.

Firstly, we will discuss characteristics of participants. Savill et al. (2017) stated that *gender* is an important moderator for the effectiveness of body psychotherapy on negative symptoms in schizophrenia. They performed a secondary analysis with the data of a large multicenter randomized controlled trial by Priebe et al. (2016), the so-called “NESS paper.” The interaction between gender and treatment allocation as a predictor of outcomes was examined in 275 participants (72 women and 203 men) randomized to either a body psychotherapy or a Pilates group (for a critique of the study, particularly its control group selection, see the last paragraph of the discussion on the need for mechanism studies). Negative symptoms in schizophrenia “were found to significantly decrease in women randomized to the body psychotherapy condition in comparison to Pilates, while no such effect was detected in men” (Savill et al., 2017, p. 1). To approach this issue, we also assessed percentages of female participants in intervention and control groups in our meta-analysis. We conducted a sensitivity analysis using the total percentage of female participants and the relation between female and male participants of intervention group and control group as a moderator. We found no significant influence of gender. This type of analysis has low power, which means that there is a high risk for a β-error. Thus, future research needs to investigate whether gender is an important moderator of effects. Further potential participant-related factors are self-efficacy or outcome expectations (Murrock and Madigan, 2008), attitude toward intervention (treatment adherence, motivation), and previous dance experiences. Regarding characteristics of DMT interventions, there is a high variety of methods and therapeutic styles, because only a few of the trials included manualized implementation of treatment (Martin et al., 2016; Priebe et al., 2016). Dance instructors used many different dance styles and teaching approaches. Although it was not possible in our meta-analysis, it would be useful to investigate differences between the effects of several methods and styles in the DMT and dance spectrum, in order to gain more knowledge about differential indications (Koch, 2019) and therapeutic factors. In her meta-analysis, Peters (2012) found *qualification of therapist* to be a significant moderator of effects. Concerning environmental factors, she discussed the influence of *social support*. Murrock and Madigan (2008) found that social support from friends mediated the effect between culturally specific dance and lifestyle physical activity. Finally, as in most therapeutic interventions, the relationship between the therapist and the participant is important (Grawe et al., 1994; Wampold and Irmel, 2015). As previously mentioned in the Introduction, the relationship between participants (group cohesion) is also assumed to be an important mediator of effects (Schmais, 1985, 1998; Yalom, 1985) in interventions that are conducted in a group setting.

It is always difficult to observe *long-term effects* in meta-analyses. In our sample, only eight of the included trials reported follow-up data (mean period: 22 weeks). The analyses yielded various results, but most effects remained constant or increased. Regarding the encouraging follow-up findings, we assume that DMT and dance interventions have the potential to initiate a learning process (body access, interoception, insight) that might instigate positive changes several months after the intervention. Additionally, it is plausible that the permanence of effects depends on the participants' behavior after the intervention, such as revising what they have learned, continuing dance or movement classes, or maintaining contact with the other participants. Further research needs to follow to investigate long-term effects and their moderators.

### Limitations

One of the biggest limitations of our present study was the heterogeneity of results, which occurred due to mild inclusion criteria and various study characteristics of the included trials. The heterogeneity mostly concerned the dance intervention studies, whereas the DMT studies were rather homogeneous. Heterogeneity was caused, for example, by dissimilarities in methodological designs of studies, methods of interventions, and sample characteristics. Besides that, we observed effects on several different health-related psychological outcomes. For this reason, we employed a random-effect model, which considers that not all studies measure the same effect. Consequently, we obtained broad 95% confidence intervals, meaning that the “true effects” could also be much larger or smaller than the weighted mean effect sizes reported in this paper (e.g., some of the confidence intervals varied between no effect and medium effects or small effects and large effects). Such results are less conclusive. This leads to a typical critique of meta-analysis as “*comparing apples and oranges*” (Sharpe, 1997), which means that non-comparable outcomes are unjustifiably compared with each other. Yet, in cases where there is not a lot of evidence, it might be a useful start, if we want to obtain information about “fruits”—to stay in the metaphor. However, we should be aware that we lose specific information about discrete sorts of fruits and mostly create a starting point to generate useful hypotheses for future, more specific secondary analyses and primary trials.

Our meta-analysis is one of the first in the field to conduct *sensitivity analysis*, which is one approach to deepen the understanding about the sources of heterogeneity. Nevertheless, it is impossible to detect all important factors and to draw firm conclusions about causal relationships between those factors. The strength of our paper is that it provides a broad overview of current research on the therapeutic use of dance as an orientation for researchers (summarize findings, identify explanatory variables, help identifying research gaps and develop research questions, control standards of research). It informedly transfers knowledge about the effectiveness of DMT and dance interventions to practitioners, clients, and public decision-makers. However, the disadvantage of broad analyses is that we only obtain results for a rough orientation. It is the assignment of secondary analyses with more narrow research questions (such as provided in Cochrane Reviews for single clinical populations) to gain sharp and more detailed knowledge about the effectiveness of DMT and dance interventions and the interdependency with contributing contextual factors.

Another issue is the so-called “garbage-in–garbage-out problem,” meaning that the results are less conclusive if we include primary outcome trials with poor methodological quality. In the present study, we also included studies with considerable methodological constraints (e.g., small *N*, no randomization, high dropout, deficient report of implementation or statistics, conflicts of interest). The most important question is how much the methodological constraints may systematically bias the results reported in this paper (the weighted medium effect sizes). We approached the methodological variety of primary trials with sensitivity analysis. There was a tendency that more outliers, especially with large effect sizes, were studies with more severe methodological constraints. “Higher vs. lower risk studies” was a moderator of effects on a *p* < 0.1 level. The medium effect size in “lower risk studies” was *d* = 0.48 (*p* < 0.001, *I*^2^ = 8.68%), which is slightly lower and more homogeneous than the overall weighted mean effect size (*d* = 0.60, *p* = 0.001, *I*^2^ = 72.62%). This might be a hint that studies with more significant methodological constraints tend to overestimate the effects of DMT and dance. Since all of these problems were more pronounced in the dance intervention studies, one resulting recommendation is to separate DMT and dance intervention studies in the next general meta-analysis.

One important source of bias might be the “Rosenthal effect” or *expectancy effect*, which means that the expectations of the researcher are subtly communicated to the participants. By guessing the goal of the research, participants try to comply with its assumed goal (Rosenthal, 1966). Thus, self-report-questionnaires or observation rating scales are more prone to bias than cognitive, physiological, or motor test, which are somewhat less subjective, but still reactive (note that the reactiveness of measurement type ranked differently in our study as indicated above). Furthermore, it is possible that researchers tend to analyze, interpret, and report results in favor of positive effects, because of their own expectancies or potential conflicts of interest.

Another critical element that might contribute to the fact that higher-risk studies yielded larger effect sizes could be *publication bias*. One criterion of methodological quality of study was sample size. If there is publication bias, smaller studies yield larger effects, on average, because smaller studies, which did not detect positive effects, remained unpublished. In the distribution of our sample of primary trials, there was a small tendency for publication bias, which did not affect our results significantly (see Methods section). Furthermore, smaller studies yielding larger effects could also be explained by the fact that, in smaller samples, the intervention was more tailored to the individual and therefore more focused.

Apart from quality of included trials, *quantity of studies* is also a matter in meta-analysis. Compared to Koch et al. (2014), we obtained larger analysis clusters; however, especially for interpersonal skills, cognitive skills, and (psycho-)motor outcomes, more research is needed to obtain more meaningful results. The advantage of a bigger sample per outcome would be that more homogeneous clusters could be considered and addressed with sensitivity analysis. In addition, several included trials were conducted by the same research group (total: 7 of 41 trials by Koch et al., 2019), which is a threat to external validity. This is particularly relevant, when it comes to analysis of clusters (DMT group: 7 of 21 trials, clinical outcome cluster: 4 of 12 trials, interpersonal skills trials: 2 of 6 studies by the research group of Koch et al., 2019).

Finally, there is a *general discussion* on the issue of whether quantitative analyses are the appropriate means to evaluate the therapeutic use of dance. Borg (1993) stated that the dilemma of scientific (positivistic) research in behavioral sciences is that it applies traditional concepts of physical science to the study of living organisms, although living organisms are far more complex than physical objects of study. The authors imply that naturally dynamic, interdependent networks of factors involved in psychological phenomena are sometimes hard to detect when we apply concepts of causality, predictability, and scientific reducibility. This argument is underlined by a vivid discussion on the ecological validity of studies reducing complex processes, such as an aesthetic experience or the impact of art perception and production on health, into its single components (see e.g., Christensen and Jola, 2015). While quantitative methods are helpful for generating facts and explanations, qualitative methods might be more suitable for meaning-making and understanding of such (Berrol, 2000). In order to overcome the gaps between a non-linear reality and linear means of investigation, and to generate new scientific insights, quantitative research ought to be applied together with qualitative research, ideally in mixed-methods designs that reflect the epistemological background assumptions of the studied processes.

Hervey (2000) takes the discussion a step further by stating that *artistic inquiry* is needed to adequately reflect the results of such process-oriented domains as DMT. She introduces the concept of artistic inquiry, as part of *arts-based research* to DMT, which implies the use of the respective art form (dance) not only as an intervention to help the recipient but also as a form of data assessment, analysis, and presentation that aims at answering the research question. Leavy's textbook (2017) is the basic source for arts-based research today, providing a terminological and historical overview and best practice examples of arts-based research. Best practice examples of specific and particularly well-described arts-based research methods are Jola's embodied neuroscience (Jola, 2013), and Eberhard's aesthetic answering (Lange et al., in press), both participatory approaches with the researcher in an active embodied role, diving into non-verbal processes to inform and answer the research question. However, at this point, final recommendations about arts-based research as a method are difficult, because the field is young and in a dynamic development (Leavy, 2017). Generally, non-verbal methods employing DMT or dance may detect changes in psychological outcomes that are not necessarily accessible with traditional methods and thus create innovative knowledge.

### Recommendations for Future Research

This meta-analysis shows that quantitative research on the therapeutic use of dance is augmenting. However, there is still an urgent quest for more trials with rigorous standards in respect to the chosen way of research (quantitative, qualitative, arts-based). Quantitative trials should consider larger sample sizes, randomized controlled designs, and active control groups, which compare DMT and dance interventions to other psychotherapeutic interventions with existing knowledge about therapeutic mechanisms (different types of psychodynamic therapies, cognitive behavioral therapy, pharmacological therapy). Furthermore, a detailed description of the intervention and its implementation is necessary (e.g., for replications). To avoid bias, there should be as much blinding as possible involved in the process. While complete blinding is not possible in therapy studies (and should thus not be part of the quality assessment of therapeutic trials to add this critique here), blinding of the randomization process and the accessor should be standard. Researchers ought to employ assessment tools that are least sensitive to expectation effects (e.g., standardized tests, psychophysiological measurement). All relevant treatment conditions should be reported in as much detail as possible (e.g., sample characteristics, characteristics of therapists, dropouts, structure and content of the treatment, other therapies provided, and interim circumstances, i.e., all “external” events that occurred in the time of the treatment, such as change of partner or job, etc.). Statistical results should be reported in detail including results that were not in line with the central hypotheses of the papers and descriptive statistics (means, standard deviations, and sample sizes in each group). To provide information on the impact of a treatment, long-term effects must be considered. Therefore, we highly recommend including follow-up assessment in intervention studies on the therapeutic use of DMT and dance.

In order to obtain more comparable research across the globe, there should be more communication between researchers, and they should strive for international standards. We recommend researchers conducting future meta-analyses to include more precise assessment of risk of bias, than was possible here. In addition, systematic analyses of moderators of effects should be performed in the future. Because we observed many dissimilarities between DMT and dance intervention studies, we recommend analysing those two types of studies separately in future studies.

Finally, to draw conclusions for practice, there is a need to complement quantitative research inquiry with qualitative and arts-based research (best in mixed-methods designs, reflecting the epistemological framework) and with clinical mechanism studies.

### Mechanism Research Needs to Inform Outcome Research

The urgent need for mechanism studies (Kazdin, 2007) and their interdependency with outcome studies shall here be exemplified with the debate around the included “NESS paper” (Priebe et al., 2016). Priebe et al. (2016) tested the decrease of negative symptoms in patients with schizophrenia after movement therapy (i.e., body psychotherapy, BPT, conducted by dance movement therapists; for definition of these terms, see Martin et al., 2016) in a randomized sample of *N* = 275 participants. They found that “the adjusted difference in negative symptoms was 0.03 (95% CI −1.11 to 1.17), indicating no benefit from body psychotherapy. Small improvements in expressive deficits and movement disorder symptoms were detected in favor of body psychotherapy. No other outcomes were significantly different.” With the interpretation of these findings, the authors question other relevant studies of the field, including their own earlier work (see Röhricht and Priebe, 2006; Lee et al., 2015; and Martin et al., 2016—the latter a study from the same year, employing the same treatment manual (Röhricht and Papadopolous, unpublished). with a TAU control group, showing a significant reduction of negative affect after DMT (measured with the SANS). The NESS paper's data were mixed though, and further analyses in the secondary trial of Savill et al. (2017) have shown that the null effect discussed in the NESS paper was only true for men, not for women after the intervention.

Three arguments call for the necessity of a reappraisal of the NESS paper: the problematic domain overgeneralization from DMT/BPT to all arts therapies (domain and terminology-related aspect), the control group selection (DMT/BPT vs. Pilates), and the selective reporting and shortfall in conclusions, which do not appropriately reflect what is evidenced in the data (e.g., from the measures of negative symptoms, the PANSS showed no significant difference between groups, whereas the CAINS did; SANS, PANSS, and CAINS are all standardized observational measures to assess positive and negative symptoms of schizophrenia). In the context of the mechanism problem, we will merely discuss the second argument here.

Active control groups are recommended for most studies by the increasing standards of the evidence-based medicine. Priebe et al. (2016) thus tried to implement a suitable active control group. From the perspective of DMT though, with its present pronounced research on therapeutic mechanisms, Pilates is not a suited control group to DMT. Both interventions, DMT/BPT and Pilates, employ methods that are suited to increase body awareness. In Pilates, the torso is the focus of the work, the muscle tone is actively controlled and altered in specific regions of the torso, the muscles are strengthened and stretched, and the practice includes breath work for bringing the movements of the torso in resonance with the breath. Pilates had the goal of addressing the trinity of body, mind, and spirit in a holistic way (“Return to Life through Contrology,” Pilates and Miller, 1945). In DMT theory, the torso is the seat of the emotions, and the breath brings the emotions to the fore: on the basis of DMT core knowledge, the work with the torso and breath is the direct pathway to sensation, experience, and expression of emotions (e.g., Caldwell, 1996). Thus, the resulting null findings on the PANSS are not surprising. While we know very little about the mechanisms of BPT and DMT, we know even less about the mechanisms of Pilates and other body practices. Thus, it is very difficult, if not impossible, to select suitable active control groups for DMT studies, without any knowledge of either intervention's main mechanisms. This example shows that good outcome research needs scholarly mechanisms research (Hayes, 2013) and that there is a strong interdependency between these two types of clinical research.

Because mechanisms of DMT/BPT are not well-researched, and even less so mechanisms of Pilates, both the experimental group and the control group may have experienced similar working mechanisms, which may have caused the inconclusive results. As long as the major mechanisms of these therapies remain unclear, it is hard to draw any valid conclusions from the according outcome research. Thus, the effect of DMT/BPT on the reduction of negative symptoms needs to be investigated with a range of control groups. Primarily therapeutic mechanisms of DMT/BPT and potential control interventions, respectively, need to be further investigated, before conducting another primary study of the scope of the NESS paper. With this paragraph, we hope to have illustrated the urgent need for mechanisms studies due to their intricate interdependency with outcome research.

## Conclusion

In conclusion, the results of our meta-analysis suggest that therapeutic use of dance potentially affects various health-related psychological outcomes. In total, there was a medium significant overall effect based on heterogeneous results. However, since *type of intervention* was a significant source of heterogeneity, we explored trials on DMT and trials on dance interventions in two separate groups. We found empirical evidence that DMT consistently and with a high homogeneity improved affect-related psychological conditions by decreasing anxiety and depression levels, and increased quality of life and cognitive skills. Concerning interpersonal skills, the effect reached the *p* < 0.1 level. More high-quality primary studies need to be conducted and included into meta-analyses to expand the evidence. Dance intervention studies consistently improved motor skills, while findings for the other outcomes had a high heterogeneity. Results of this meta-analysis suggest that DMT and dance interventions improve clinical outcomes, cognitive outcomes, and (psycho-)motor outcomes. The high variability of results, especially in the dance cluster, needs further attention. Moreover, this study contributes initial findings that DMT and dance interventions have persistent long-term effects. These encouraging results are limited by methodological shortcomings of the primary studies. Further research is needed that expands on the evidence of effects of DMT and dance interventions on health-related psychological outcomes.

## Author Contributions

SK conceived, planned, co-wrote and revised the study and supervised the Master's (RR, KT) and doctoral level students (LM, JB). LM and KT did the systematic literature search. LM, KT, and SK did the hand search. RR and AB planned and implemented the methodological approach, AB supervised the methodology of the study. KT, JB, and RR organized the results. RR analyzed the results and wrote the first draft. All authors contributed to the paper and revised it into its final version.

### Conflict of Interest Statement

The authors declare that the research was conducted in the absence of any commercial or financial relationships that could be construed as a potential conflict of interest.

## Footnotes

^3^Creative Arts Therapies (European term: Arts Therapies; Karkou and Sanderson, 2006) consist mainly of art therapy, music therapy, dance movement therapy, drama therapy, and poetry therapy/expressive writing.
