# Supplementary material for: Effects of Dance Movement Therapy and Dance on Health-Related Psychological Outcomes. A Meta-Analysis Update
Source: Front Psychol. 2019 Aug 20;10:1806. doi: 10.3389/fpsyg.2019.01806 (PMC6710484; doi:10.3389/fpsyg.2019.01806)
Supplement: Supplementary file 1 [file Data_Sheet_1.docx]

Supplementary material

Appendix A: List of abbreviations and symbols

List of Abbreviations

| Abbreviation | Explanation |
| --- | --- |
| ADHD | attention deficit hyperactivity disorder |
| ASD | autism spectrum disorder |
| BDI | Beck Depression Inventory |
| BSI | Brief Symptom Inventory |
| CG | control group |
| CI | confidence interval |
| *d* | Cohen's *d* |
| df | degree of freedom |
| DMT | dance movement therapy |
| e.g. | exempli gratia (for example) |
| EG | experimental group |
| ES | effect size |
| HADS | Hospital Anxiety and Depression Scale |
| *I*² | *I*-square (percentage of heterogeneity) |
| ICD 10 | International Classification of Diseases vol. 10 |
| *k* | sample size (number of studies) |
| M | Mean |
| Max | maximum |
| Min | minimum |
| *N* | sample size (number of participants) |
| *p* | p value |
| SE | standard error |
| SMD | standardized mean differences |
| STAXI | State Trait Anger Expression Inventory |
| β | beta (gradient parameter) |
| TAU | Treatment as usual |
| UPDRS | Unified Parkinson Disease Rating Scale |
| Y | Years |

List of symbols

| Symbol | Explanation |
| --- | --- |
| $d$ | Cohen’s *d* |
| $d_{i}$ | Cohen’s *d* (mean per study) |
| $d_{c}$ | Cohen’s *d* (mean per analysis-cluster per study) |
| $d_{c,i}$ | Cohen’s *d* (mean per analysis-cluster) |
| $N_{i}$ | sample size (number of participants) per study |
| $N_{iIG}$ | sample size in intervention group per study |
| $N_{iCG}$ | sample size in control group per study |
| $SD$ | standard deviation |
| $SDpooledpre$ | pooled standard deviation before intervention |
| ${SD}_{IGpre}$ | standard deviation in intervention group before intervention |
| ${SD}_{CGpre}$ | standard deviation in control group before intervention |
| $\omega_{i}$ | weight per study |

Appendix B: Differentiated reasons for exclusion of the last 14 studies

| Excluded studies from between 2012 and March 2018 | Reasons for exclusion |
| --- | --- |
| Alotaibi, Karkou, Van Der Linden, & Irvine, 2017 | Data insufficient for effect size calculations |
| Alrazain, Zubala, and Karkou, 2018 | Data insufficient for effect size calculations |
| Burzynska, Jiao, Knecht, Fanning, Awick, and Kramer, 2017 | Data insufficient for effect size calculations |
| Cross, Flores, Butterfield, Blackman, & Lee, 2012 | Intervention did not fulfill inclusion criteria (no active dancing, just observation) |
| Duim, do Nascimento, & da Silva, 2015 | Data insufficient for effect size calculations |
| Ho, 2016b | Data insufficient for effect size calculations (Qualitative Data) |
| Koch, Juenger, Kelbel, Kolter, Sattel, & Fuchs, 2017 | Data insufficient for effect size calculations |
| Lewis, Annett, Davenport, Hall, & Lovatt, 2014 | Control group did not fulfill inclusion criteria (not comparable to intervention group) |
| Mateos-Moreno & Atencia-Doña, 2013 | Intervention did not fulfill inclusion criteria (various tools of creative art therapy) |
| Meyer DeMott, Jakobsen, Wentzel-Larsen, & Heir, 2017 | Intervention did not fulfill inclusion criteria (various tools of creative art therapy) |
| Savill, Orfanos, Bentall, Reining**­**haus, Wykes, & Priebe, 2017 | Same data set as Priebe, Savill, et al. (2016) |
| Stück & Villegas, 2017 | Data insufficient for effect size calculations |
| Vancea, 2013 | Data insufficient for effect size calculations |
| Van Westrhenen, Fritz, Vermeer, Boelen, & Kleber, 2017 | Intervention did not fulfill inclusion criteria (various different and mixed creative art therapy) |
